# Supplementary material for: Automated Identification and Localization of Hematopoietic Stem Cells in 3D Intravital Microscopy Data
Source: Stem Cell Reports. 2015 Jun 25;5(1):139–53. doi: 10.1016/j.stemcr.2015.05.017 (PMC4618449; doi:10.1016/j.stemcr.2015.05.017)
Supplement: Document S1. Supplemental Experimental Procedures, Figures S1–S3, and Tables S1–S4 [file mmc1.pdf]

Figure S1.

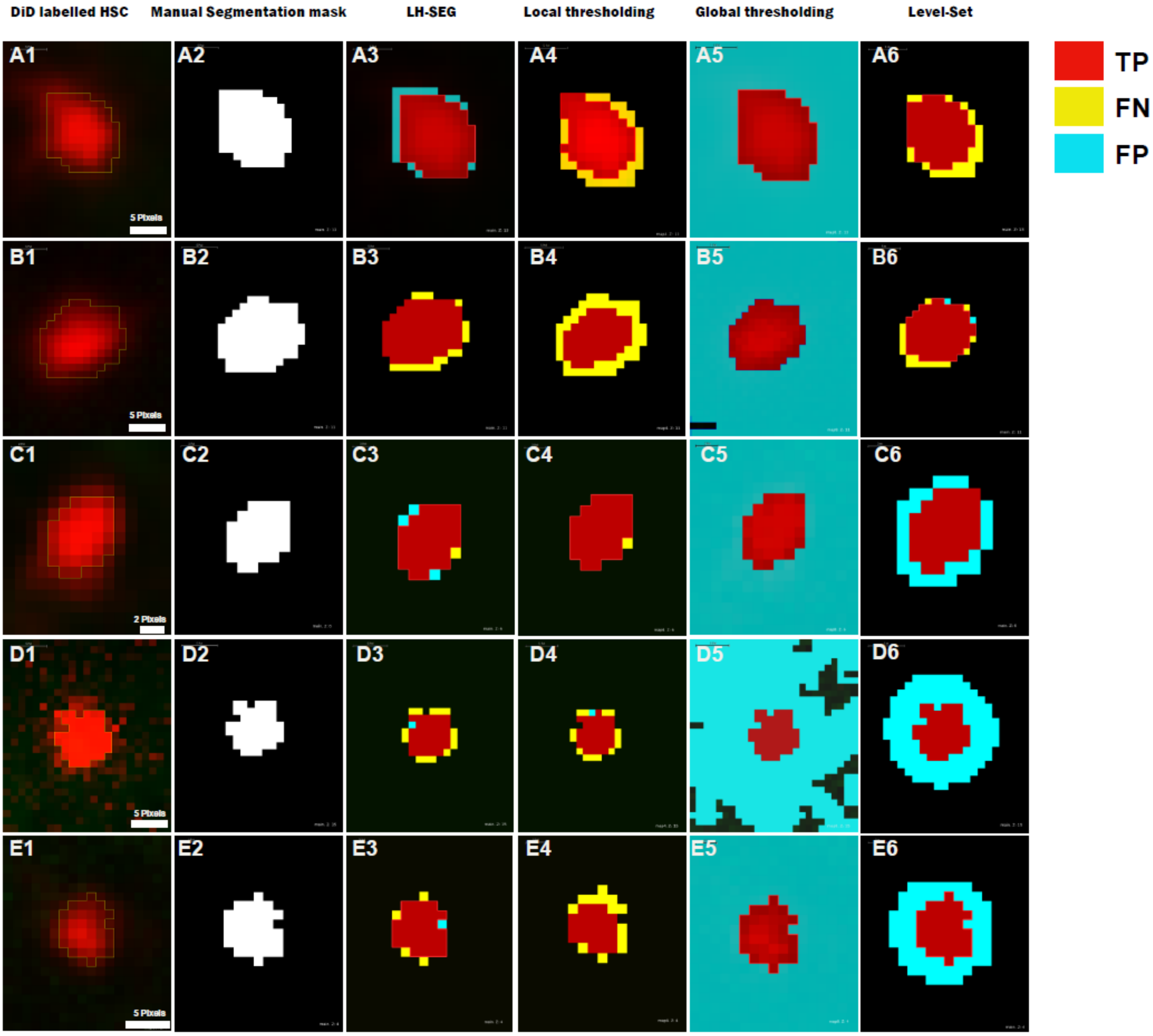

DiD labelled HSC
Manual Segmentation mask
LH-SEG
Local thresholding
Global thresholding
Level-Set
A1
A2
A3
A4
A5
A6
B1
B2
B3
B4
B5
B6
C1
C2
C3
C4
C5
C6
D1
D2
D3
D4
D5
D6
E1
E2
E3
E4
E5
E6
5 Pixels
5 Pixels
2 Pixels
5 Pixels
5 Pixels
TP
FN
FP

Figure S2.

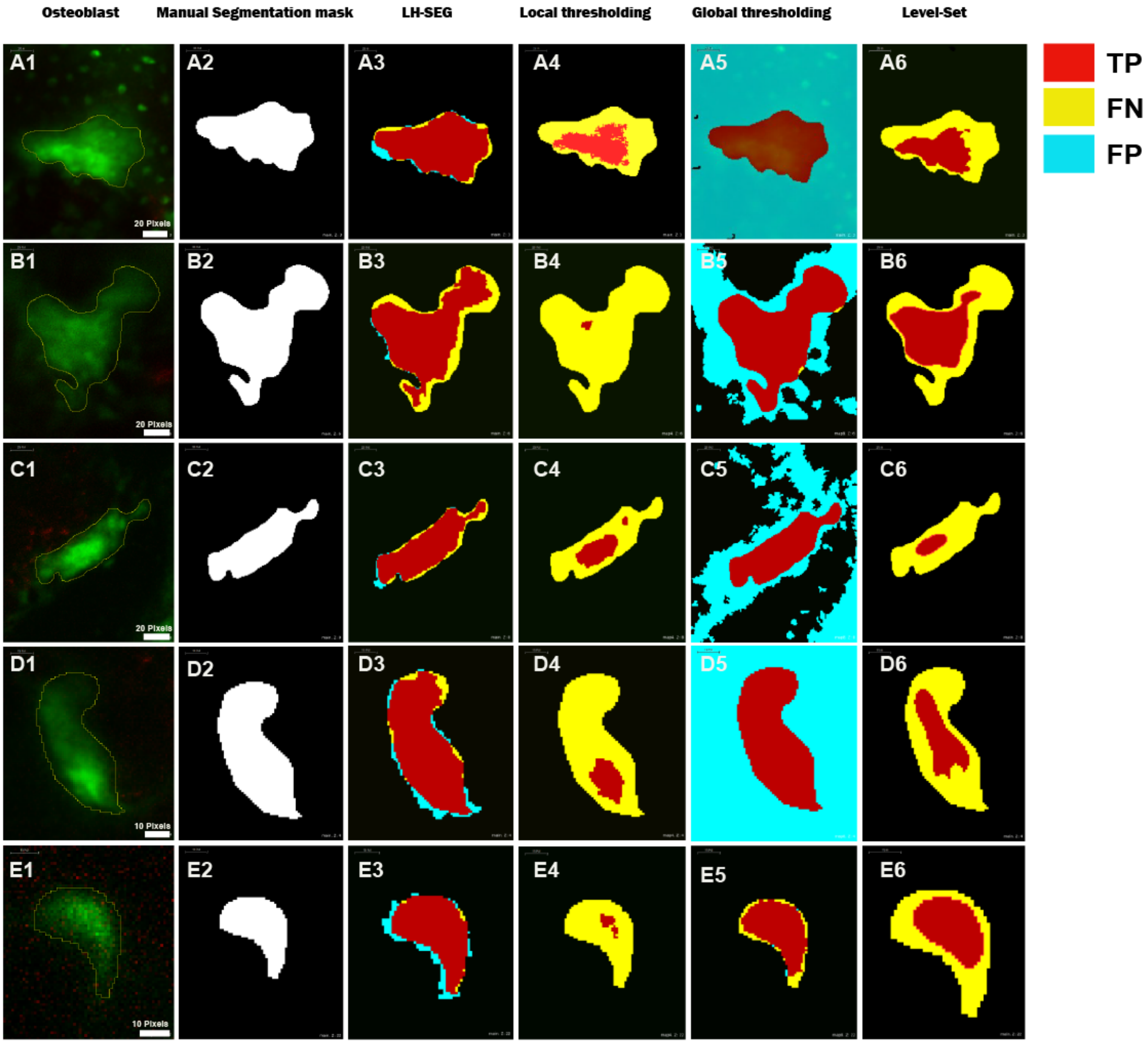
Osteoblast
Manual Segmentation mask
LH-SEG
Local thresholding
Global thresholding
Level-Set
A1
A2
A3
A4
A5
A6
B1
B2
B3
B4
B5
B6
C1
C2
C3
C4
C5
C6
D1
D2
D3
D4
D5
D6
E1
E2
E3
E4
E5
E6
20 Pixels
10 Pixels
TP
FN
FP

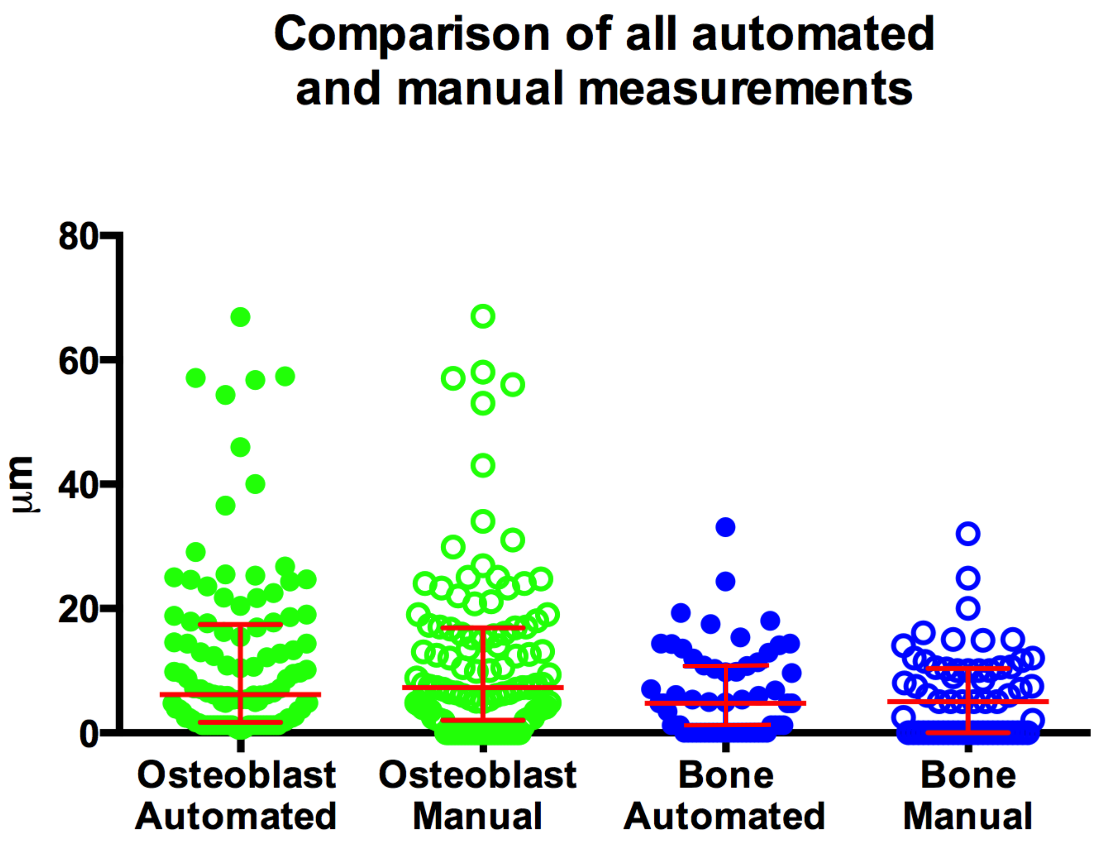

Comparison of all automated
and manual measurements
80
60
40
20
0
µm
Osteoblast
Automated
Osteoblast
Manual
Bone
Automated
Bone
Manual

Figure S1. Comparison of the manual benchmarking segmentation of DiD-labeled objects against the performance of our proposed Local Homogeneity segmentation method (LH-SEG), local adaptive thresholding, global thresholding and level set segmentation. (A1-E1) Examples of DiD-labeled objects exhibiting different intensity, edge morphology and neighborhood intensity characteristics. (A2-E2) Manual segmentation benchmarking masks of the DiD-labeled HSC. (A3 - E6) Segmentation masks obtained with the methods tested (ours (LH-SEG): A3-E3; local adaptive thresholding: A4-E4; global thresholding: A5-E5; level set segmentation: A6-E6). Red pixels are in both benchmark and tested mask (true positive TP); yellow pixels are in the benchmark but not in the test mask (false negative FN); blue pixels are in the test but not in the benchmark mask (false positive FP). The proportions of true positive, false positive and false negative pixels were used to evaluate the performance of each segmentation method. Pixel size is 0.8 µm for (A, B, D, E) and 1.2 µm for (C), Related to Performance evaluation; Segmentation accuracy section.

Figure S2. Comparison of the manual benchmarking segmentation of GFP$^+$ osteoblastic cells against the performance of our proposed Local Homogeneity segmentation method (LH-SEG), local adaptive thresholding, global thresholding and level set segmentation. (A1-E1) Examples of GFP$^+$ osteoblastic cells exhibiting different intensity, edges morphology and neighborhood intensity characteristics. (A2-E2) Manual segmentation benchmarking masks of the GFP$^+$ osteoblastic cells. (A3 - E6) Segmentation masks obtained with the methods tested (ours (LH-SEG): A3-E3; local adaptive thresholding: A4-E4; global thresholding: A5-E5; level set segmentation: A6-E6). Red pixels are in both benchmark and tested mask (true positive TP); yellow pixels are in the benchmark but not in the test mask (false negative FN); blue pixels are in the test but not in the benchmark mask (false positive FP). The proportions of true positive, false positive and false negative pixels were used to evaluate the performance of each segmentation method. Pixel size is 0.8 µm for (A, B, C, E) and 1.2 µm for (D), Related to Performance evaluation; Segmentation accuracy section.

Figure S3. Comparison of automated and manual (benchmark) 3D measurements. All identified HSCs across the 10 datasets were analyzed in 3D to identify the shorter distance between the cells and either osteoblasts (green) or bone (blue). In the graph, each circle represents a single analyzed cell. Automated measurements (full circles) and manual, benchmark measurements (empty circles) were very similar (see Figure 6 for error details) and provided a distribution patterns of (p> 0.75, Mann Whitney test; n= 102 measurements to

osteoblasts and 55 measurements to bone from 10 and 5 independent datasets, respectively). Related to Performance evaluation; 3D localization accuracy section.

Movie S1. Representative example of intravital microscopy data. A 3D stack containing DiD-labeled objects (red), GFP+ osteoblastic cells (green) and SHG signal of bone collagen fibers (blues) is shown as a z-series of 22 slices starting from top (bone) to bottom (deep bone marrow tissue). The z step size is 5 um, the field of view is 512 um2, Related to Results; Variability of Intravital Microscopy Datasets section.

Table S1. The 10 datasets used to develop and evaluate LH-SEG, HSC recognition and 3D measurements. Each dataset corresponds to a single mouse analysed by intravital microscopy and contains a variable number of 3D stacks (fields of view) acquired at varying magnification (213-620 in μm^2) and with step sizes of 1 or 5μm. All datasets included GFP and DiD signal an some included also second harmonic generation (SHG) signal from bone collagen. All datasets were from intravital microscopy of HSCs, however donor mice were either control (wilt type, WT) or infected (inf) animals, recipient mice were either wild type (WT) or col2.3Dkk1 (Dkk) transgenic, and the HSCs were FACS purified using either the LKS CD150 CD48 or LKS CD34 Flk2 combinations of markers, Related to Results; Variability of Intravital Microscopy Datasets section.

Table S2. Processing times and number of segments obtained for multi-resolution segmentation depending on the value of parameter α. Multi-resolution segmentation, the first step of LH-SEG, was run one the same 3D stack (dataset 10, field of view 4) using different values for parameter α for each channel (DiD signal, osteoblastic cells and bone collagen). Smaller α values led to larger numbers of segments and longer processing times, Related to Results; Local Heterogeneity-based Image Segmentation (LH-SEG) section.

Table S3. Summary of the optimized parameters used for LH-SEG. DiD, GFP osteoblast and SHG bone collagen signals required each their own set of optimized parameters, however the same parameters could be used for all the images throughout the datasets analysed. The first step of LH-SEG required optimization of parameter α, and the second step of LH-SEG required optimization of parameters $T_{\bar{\Delta}_k}$ and d . The values selected for each parameter and

each channel/object are indicated, Related to Results; Local Heterogeneity-based Image Segmentation (LH-SEG) section.

Table S4. The intensity, morphological and textural features used to train the classifier. List of all features used to train the classifier, grouped by their type. The classifier itself selected the features highlighted in red as the discriminative ones to differentiate HSCs (in two classes) from non-specific DiD signal, Related to Results; Machine Learning Classification of HSCs section.

Segment-Classify-Measure-distance-3D.dcp is the source code file, ready to use in Definiens environment for quantifying HSCs, osteoblast and bone. For quantifying other bone marrow components follow the instructions provided in the supplemental data sections: Guidance on how to optimise LH-SEG parameters, Optimised LH-SEG parameter values for the experiments described and Table S3.

Table S1. The 10 datasets used to develop and evaluate LH-SEG, HSC recognition and 3D measurements.

Data type	Data set No.	Fields of view	Z (no. of Slices)	X,Y μm^2	Step size μm	Channels analysed	Type of experiment	HSCs Markers
3D Stack	1	12	37-88	213-396	1	DiD, GFP	WT into WT	LKS,CD150+, CD48-
3D Stack	2	9	62-92	248-378	1	DiD, GFP	WT into Dkk	LKS, CD150+, CD48-
3D Stack	3	10	45-94	268-405	1	DiD, GFP	WT into WT	LKS, CD150+, CD48-
3D Stack	4	8	57-102	308-450	1	DiD, GFP	WT into Dkk	LKS, CD150+, CD48-
3D Stack	5	10	14-29	620	5	DiD, GFP, SHG	WT into WT	LKS, CD34-, Flk2-
3D Stack	6	6	17-28	620	5	DiD, GFP, SHG	WT into WT	LKS, CCD34-, Flk2-
3D Stack	7	10	13-35	620	5	DiD, GFP	WT into WT	LKS, CD150+, CD48-
3D Stack	8	10	12-40	620	5	DiD, GFP,SHG	WT into WT	LKS, CD150+, CD48-
3D Stack	9	10	20-27	620	5	DiD, GFP.SHG	Inf into WT	LKS, CD34-, Flk2-
3D Stack	10	10	17-25	620	5	DiD, GFP,SHG	Inf into WT	LKS, CD34-, Flk2-

Table S2. Processing times and number of segments obtained for multi-resolution segmentation depending on the value of parameter α.

Cellular component	Scale parameter	Processing time	Number of segments
DiD labeled HSC	α = 3	01.08 mins	274.143
DiD labeled HSC	α = 8	00.37 mins	56,449
GFP+ osteoblastic cells	α = 3	8.47 mins	492 ,562
GFP+ osteoblastic cells	α = 10	1.06 mins	56,516
SHG bone collagen signal	α = 3	6.16.80 mins	501,339
SHG bone collagen signal	α = 8	01.09.30 mins	71,320

Table S3. Summary of the optimized parameters used for LH-SEG.

Object type	Multi-resolution optimized parameter	MDN optimized parameters	
HSCs	α =8	$T_{\bar{\Delta}_k} \geq 68$	$d = 30$
Osteoblast	α =10	$T_{\bar{\Delta}_k} \geq 8$	$d = 60$
Bone	α =8	$T_{\bar{\Delta}_k} \geq 4$	$d = 70$
Vasculature	α =8	$T_{\bar{\Delta}_k} \geq 4$	$d = 40$
Nestin	α =8	$T_{\bar{\Delta}_k} \geq 3$	$d = 60$
mTmG progenitors	α =8	$T_{\bar{\Delta}_k} \geq 10$	$d = 30$
Macrophages	α =8	$T_{\bar{\Delta}_k} \geq 68$	$d = 30$

Table S4. The intensity, morphological and textural features used to train the classifier. Features selected by the classifier as the most discriminative features are highlighted in red.

Feature	Feature type
Brightness	Intensity
Standard deviation	Intensity
Area	Morphological
Border length	Morphological
Length	Morphological
Length/width	Morphological
Number of pixels	Morphological
Rel. Border to image Border	Morphological
Thickness	Morphological
Volume	Morphological
Width	Morphological
Asymmetry	Morphological
Compactness	Morphological
Density	Morphological
Elliptic fit	Morphological
Radius of largest enclosed ellipse	Morphological
Radius of smallest enclosed ellipse	Morphological
Rectangular fit	Morphological
Roundness	Morphological
Shape index	Morphological
GLCM Homogeneity	Textural
GLCM Dissimilarity	Textural
GLCM Entropy	Textural
GLCM Ang. 2nd Moment	Textural
GLCM Mean	Textural
GLCM Std. Dev.	Textural
GLCM Correlation	Textural
GLDV Entropy	Textural
GLCM Contrast	Textural
GLCM StdDev	Textural

Guidance on how to optimise LH-SEG parameters:

In practice, users will have to select only three parameter for each object type (e.g. HSC, osteoblast, bone etc.) and each parameter would have to be optimized one time before starting the analysis, once optimized it should work for different image datasets given that the object types are still the same.

For each parameter users only need to provide a rough estimate of the average parameter value. The parameters are then integrated into the algorithm automatically.

Parameter 1: Multi-resolution parameter α

This parameter is mainly responsible for the selection of the segments size resulting from applying the Multi-resolution segmentation. Higher values for the scale parameter will result in larger image segments, while smaller values will result in smaller image segments. The default setting for this parameter is 8 and users are advised to use it. Smaller values will result in increased running time. A good indication to confirm the correct parameter selection will be edge detection of the regions of interests.

Parameter 2: Mean intensity difference to neighbour threshold $T_{\bar{\Delta}_k}$

The value of the threshold along with the neighbourhood size (Parameter 2) will determine which segments (resulted from the multi-resolution) are merged together to form the real edges of the object of interest. To select the parameter $T_{\bar{\Delta}_k}$ value, the users need to provide a rough estimate of the mean intensity difference to neighbour threshold. To do this the user can activate the mean intensity difference to neighbour feature available in the feature window of Definiens, select segments from the background that are close to the edges of the object of interest then select the highest value found and set it as a threshold.

Parameter 3: neighbourhood size d

The selection of the parameter d determines the size of the neighbourhood in which a particular object covers. To select a neighbourhood size, the users need to provide a rough estimate of the average object diameter size in pixels.

Optimised LH-SEG parameter values for the experiments described

- Parameter optimization for the multi-resolution segmentation parameter α for each image object category: To detect small objects such as HCSs, bone marrow micro-cavities, myeloid progenitors, nestin GFP cells and microvessels we selected a slightly smaller scale parameter $\alpha = 8$ compared to that used for larger objects such as osteoblasts $\alpha = 10$. Importantly, selecting smaller scale parameters did not result in significant edge detection improvement, but did increase computational time thus providing support for the values chosen above.

- Optimization of parameter $T_{\bar{\Delta}_k}$ to correctly segment each image object category MDN: a high threshold of $T_{\bar{\Delta}_k} \geq 68$ for HSCs and macrophages was selected to restrict the selection of segments to those with higher intensities, in contrast we select smaller threshold for tomato expressing progenitors $T_{\bar{\Delta}_k} \geq 10$ osteoblast $T_{\bar{\Delta}_k} \geq 8$, bone and vasculature $T_{\bar{\Delta}_k} \geq 4$ and nestin $T_{\bar{\Delta}_k} \geq 3$ to allow the selection of segment with more heterogonous intensities.

- Optimization of parameter d to correctly segment each image object category: for the HCSs d was based on our observation that a single HSC will most likely cover a neighborhood of 10-30 pixels, depending on the HSC size (8-12µm in diameter) (Lo Celso et al., 2009; Lo Celso et al., 2011) and the magnification settings used, therefore we set $d = 30$. The same was for progenitors and macrophages. In contrast, given that osteoblasts and bone occupy relatively larger neighborhoods, we set $d = 60$ for the osteoblast and nestin cells $d = 70$ for bone collagen and $d = 40$ for vessels.

Definition of each selected feature by the decision tree classifier

1) Roundness

The Roundness feature describes how much the shape of an image object is similar to an ellipsoid. The more the shape of an image object is similar to an ellipsoid, the lower its roundness. It is calculated by the difference of the enclosing ellipsoid and the enclosed ellipsoid. The radius of the largest enclosed ellipsoid is subtracted from the radius of the smallest enclosing ellipsoid.

2) **Length/width**

The length-to-width ratio of an image object in 3D

3) **Grey level co-occurrence matrix (GLCM) contrast**

Contrast is the opposite of homogeneity. It is a measure of the amount of local variation in the image. It increases exponentially as i, j increase.

$$\sum_{i,j=0}^{N-1} P_{i,j}(i-j)^2$$

Where i is the row number, j is the column number, $P_{i,j}$ is the normalized value in the cell and N is the number of rows or columns.

As defined in the Defineins reference book. For more information on the parameters used to calculate the features refer to the Defineins reference book.
