# Supplementary figures and images for: Automated Identification and Localization of Hematopoietic Stem Cells in 3D Intravital Microscopy Data
Source: Stem Cell Reports. 2015 Jun 25;5(1):139–53. doi: 10.1016/j.stemcr.2015.05.017 (PMC4618449; doi:10.1016/j.stemcr.2015.05.017)

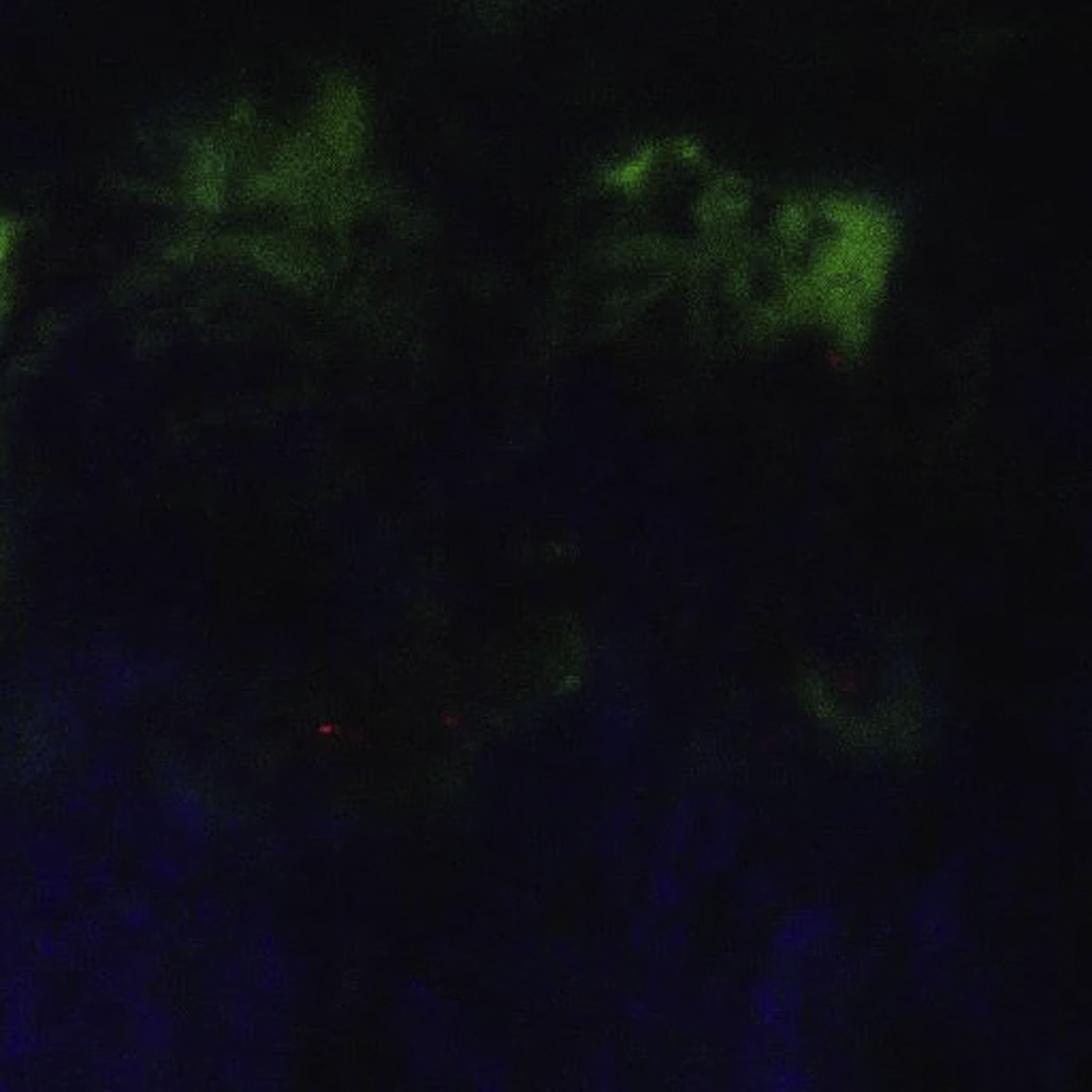

Supplement: Movie S1. Representative Example of Intravital Microscopy Data, Related to Results — A 3D stack containing DiD-labeled objects (red), GFP+ osteoblastic cells (green), and SHG signal of bone collagen fibers (blues) is shown as a z series of 22 slices starting from top (bone) to bottom (deep bone marrow tissue). The z step size is 5 μm and the field of view is 512 μm2. [file mmc3.jpg]
