# Supplementary material for: Automated Identification and Localization of Hematopoietic Stem Cells in 3D Intravital Microscopy Data
Source: Stem Cell Reports. 2015 Jun 25;5(1):139–53. doi: 10.1016/j.stemcr.2015.05.017 (PMC4618449; doi:10.1016/j.stemcr.2015.05.017)
Supplement: Document S3. Article plus Supplemental Information [file mmc4.pdf]

Stem Cell Reports
Resource
OPEN ACCESS

Automated Identification and Localization of Hematopoietic Stem Cells in 3D Intravital Microscopy Data

Reema A. Khorshed,[1,*] Edwin D. Hawkins,[1] Delfim Duarte,[1] Mark K. Scott,[1,2,3] Olufolake A. Akinduro,[1] Narges M. Rashidi,[1,4] Martin Spitaler,[2,5] and Cristina Lo Celso[1,*]
[1]Department of Life Sciences, Imperial College London, London SW7 2AZ, UK
[2]Facility for Imaging by Light Microscopy, Imperial College London, London SW7 2AZ, UK
[3]Present address: Centre for Dynamic Imaging, The Walter & Eliza Hall Institute, 1G Royal Parade, Parkville, VIC 3052, Australia
[4]Present address: The Ragon Institute of MGH, MIT and Harvard, 400 Technology Square, Cambridge, MA 02139, USA
[5]Present address: Max Planck Institute for Biochemistry, Am Klopferspitz 18, 82152 Martinsried, Germany
*Correspondence: r.khorshed@imperial.ac.uk (R.A.K.), c.lo-celso@imperial.ac.uk (C.L.C.)
http://dx.doi.org/10.1016/j.stemcr.2015.05.017
This is an open access article under the CC BY license (http://creativecommons.org/licenses/by/4.0/).

SUMMARY

Measuring three-dimensional (3D) localization of hematopoietic stem cells (HSCs) within the bone marrow microenvironment using intravital microscopy is a rapidly expanding research theme. This approach holds the key to understanding the detail of HSC-niche interactions, which are critical for appropriate stem cell function. Due to the complex tissue architecture of the bone marrow and to the progressive introduction of scattering and signal loss at increasing imaging depths, there is no ready-made software to handle efficient segmentation and unbiased analysis of the data. To address this, we developed an automated image analysis tool that simplifies and standardizes the biological interpretation of 3D HSC microenvironment images. The algorithm identifies HSCs and measures their localization relative to surrounding osteoblast cells and bone collagen. We demonstrate here the effectiveness, consistency, and accuracy of the proposed approach compared to current manual analysis and its wider applicability to analyze other 3D bone marrow components.

INTRODUCTION

Precise regulation of somatic stem cell function is essential for the survival of multicellular living organisms ranging from *C. elegans* to humans. Somatic stem cells maintain themselves while their progeny turn over and differentiate to maintain the tissue they reside in throughout life. This process is deregulated during disease and aging; therefore, increasing attention has been dedicated to understanding somatic stem cells with an aim to improve both prevention and treatment of disease. The correct functioning of somatic stem cells depends on complex and dynamic interactions with specific cellular and molecular components of the microenvironment that surrounds them (together called “niche”) (Scadden, 2014), and in vivo imaging of stem cells is an expanding and promising field that provides a unique perspective of their behavior in situ. To date, this approach has been directly responsible for generating new hypotheses on the crucial role of the stem cell niche (Ritsma et al., 2014; Rompolas et al., 2012).

Hematopoietic stem cells (HSCs) maintain the turnover of red blood cells, platelets, and immune cells. They reside in the bone marrow, where several cell types contribute to their regulation (Lo Celso et al., 2011; Morrison and Scadden, 2014). How the concerted action of multiple niche components regulates HSC fate is not clear, and understanding the localization of HSCs relative to multiple surrounding cellular and structural constituents of the bone marrow microenvironment is the first step toward solving this puzzle. Single-cell resolution intravital microscopy of fluorescently labeled HSCs and niche components allows direct observation of HSCs in mouse bone marrow. We, and others, have successfully used fluorescent dyes to label HSCs prior to transplantation to achieve their detection in vivo through the bone of anesthetized recipient mice (Köhler et al., 2009; Lo Celso et al., 2009).

The endosteal niche is a bone marrow microenvironment proximal to trabecular/cortical bone, lined by cells of the osteoblast lineage, and associated with effective HSC engraftment as well as maintenance of their long-term survival and function (Calvi et al., 2003; Kunisaki et al., 2013; Lane et al., 2011). Two-photon microscopy is essential to detect second harmonic generation (SHG) signal emitted by bone collagen and to recognize the location of bone marrow cavities and their endosteal surface (Lo Celso et al., 2011). Transgenic reporter mice expressing GFP under the control of an osteoblast-specific promoter (herein referred to as Col2.3GFP mice) (Kalajzic et al., 2002) are a powerful tool for visualizing the HSC endosteal niche. Using these and other reporter strategies, manual analysis of HSC in vivo images has provided the indication that normal HSCs localize near vasculature, endosteum, osteoblastic cells, and nestin-positive mesenchymal progenitor cells (Lane et al., 2011; Lo Celso et al., 2009; Méndez-Ferrer et al., 2010; Sanchez-Aguilera et al., 2011). However, such analysis has the following limitations: (1) it is time-consuming and, therefore, limited to measuring a few parameters; and (2) it is subject to human error, leading to

CrossMark

intra- and inter-researcher inconsistencies. We reasoned that specialized image analysis tools would simplify the biological interpretation of 3D HSC microenvironment images, and they not only would provide unbiased data analysis but also expand the number of measurable parameters with the potential to uncover new aspects of HSC biology.

Although several segmentation and classification methods have been developed for 2D (Chung and Vese, 2009; Saikumar et al., 2012; Yeo et al., 2011) and 3D datasets (Lou et al., 2014; Nandy et al., 2014; Pop et al., 2013), their application to in vivo bone marrow images is not straightforward due to the complexity of the structures of interest. In conjunction, light scattering caused by the surrounding tissue, especially by overlaying bone, limits the resolution of in vivo microscopy of bone marrow compared to that of other tissues or ex vivo techniques. To overcome these issues, we propose a local heterogeneity-based image segmentation (LH-SEG) approach that utilizes multi-resolution segmentation (Mallinis et al., 2008) and mean intensity difference to neighbor thresholding. This approach measures local morphological and intensity homogeneity and combines these values with neighborhood distance features to segment and threshold the objects of interest. To ensure reliable edge detection across objects with high-intensity heterogeneity, LH-SEG is applied on each 2D slice prior to 3D rendering.

HSCs labeled ex vivo using lipophilic membrane dyes such as 1,1′-Dioctadeciyl-3,3,3′,3′-Tetramethylindodicarbocyanine (DiD) generate a bright fluorescent signal (Lo Celso et al., 2009, 2011). However, these dyes lead to background signal from cell debris and aggregates (Progatzky et al., 2013), posing the extra challenge of filtering the dye signal to identify bona fide HSCs, a task that is not trivial even for the experienced user. In order to standardize HSC recognition, we used a machine learning protocol based on morphological and textural features to recognize and classify all segmented DiD signal. Finally, once HSCs, osteoblasts, and bone are identified, the minimum distance between each HSC and osteoblast/endosteum objects can be calculated in 3D. We used the proposed approach to examine the localization of HSCs in multiple in vivo datasets and tested its performance compared to other widely used segmentation methods as well as manual benchmarking data. We demonstrate that the method is robust and applicable to a variety of datasets that are challenging to analyze manually.

RESULTS

Variability of Intravital Microscopy Datasets

Combined confocal/two-photon microscopy allows detection of HSCs by intravital microscopy of mouse calvarium bone marrow; however, a variety of acquisition settings can be used on different days and by different users, leading to overall brighter/dimmer images and a range of field-of-view sizes and depths. We aimed to develop an image analysis protocol sufficiently flexible to deal with such variations, as it is essential to eliminate the bottleneck of data processing.

To test the applicability of our analysis protocol to a wide range of HSC in vivo datasets, we worked with ten independently acquired image datasets, exhibiting variability at both the biological and image acquisition levels. Each dataset contained 6–12 3D stacks (fields of view), including one or multiple DiD signals, originated from multiple experiments (summarized in Table S1) as follows: control HSCs (from wild-type, untreated mice) injected into Col2.3GFP recipients; control HSCs injected into Col2.3GFP × Col2.3Dkk1 double-transgenic mice (in which the same promoter drives expression of GFP and the Wnt inhibitor Dkk1, causing HSCs to lose self-renewal ability through a still unknown mechanism (Fleming et al., 2008); and infection-exposed HSCs injected into Col2.3GFP recipients (these HSCs, having become motile, interact with the bone marrow microenvironment differently than controls [Rashidi et al., 2014]).

Of the ten datasets analyzed, five (datasets 5, 6, 8, 9, and 10) contained SHG bone signal in addition to DiD and GFP signals. Movie S1 shows one example of raw data (dataset 10, field of view 4). All images were encoded at 8 bits per pixel, at a resolution of 512 x 512 pixels, but z stacks had varying sizes, with 512 pixels in the x and y dimensions corresponding to 213–620 μm and 12–102 slices acquired with a step size of 1–5 μm, with each slice corresponding to 1 voxel in the z dimension. Moreover, each dataset was generated using unique acquisition settings, from exciting laser power to detectors' gain and offset. The datasets used reflected the range of settings selected by individual researchers performing intravital microscopy experiments, and they allowed us to immediately test whether our image analysis protocol would provide reliable results in independently acquired datasets.

LH-SEG

The two main challenges faced when segmenting bone marrow in vivo images are the following: (1) the unpredictable shapes and sizes of stroma components, as osteoblasts have asymmetrical contours and cluster into irregular groups, and bone cavities vary in sizes, from microcavities to large areas occupied by bone marrow; and (2) the variable levels of fluorescence intensity, due to loss of signal with increasing depth and, in the case of osteoblasts, to the co-existence of cells expressing higher and lower levels of GFP within the same field of view and sometimes even within the same cluster. Similar challenges apply to the

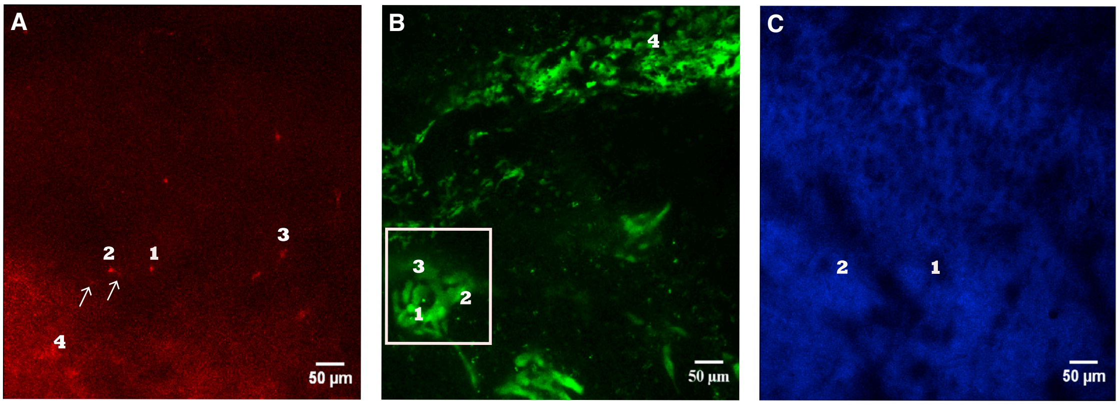

Figure 1. Examples of Raw Bone Marrow In Vivo Images

Maximum intensity projection of a 3D stack including DiD signal (A, red), osteoblastic cells (B, green), and bone collagen SHG signal (C, blue).

(A) DiD signal includes the following: (1) a single DiD-labeled HSC, (2) a DiD-labeled HSC adjacent to DiD debris (arrows), (3) DiD debris, and (4) background noise and autofluorescence.

(B) GFP-positive osteoblastic cells are highly heterogeneous and include bright (1), medium bright (2), and dim (3) relatively large, polygonal cells, and more irregular, star-shaped cells (4). The white box surrounds an osteoblast cluster containing cells of variable levels of fluorescence intensity, due to the loss of signal with increasing depth and to the co-existence of osteoblasts expressing higher and lower levels of GFP.

(C) SHG signal from bone collagen varies with depth (1). Bone cavities have variable sizes and shapes (2).

segmentation of HSCs, because, even though their shape is more homogeneous, they vary from one to another in terms of signal intensity due to their varying depth and DiD being diluted upon cell division (Figure 1).

To handle fluorescence intensity heterogeneity, we applied a convolution Gaussian blur filter with (3 × 3) kernel size (Moon, 2001), using a small kernel size to reduce intensity heterogeneity while conserving object structures. To segment the objects of interest, we developed a two-step method that recapitulates how humans identify objects based on how their intensities compare to other objects in the surrounding neighborhood. We named this segmentation approach LH-SEG. To minimize artifacts due to depth-dependent loss of signal, this method was applied to each 2D slice.

Multi-resolution segmentation is the first step of LH-SEG. It breaks highly heterogeneous images into a number of smaller segments, each of them more homogeneous in terms of both texture and shape (Baatz and Schäpe, 2000). It starts with a single pixel and iteratively merges further pixels in a number of loops as long as a threshold of homogeneity is not exceeded locally, within the segment.

The homogeneity threshold depends on the value of the scale parameter α, which reflects a combination of shape and texture homogeneity (Baatz and Schäpe, 2000). The selection of the value of this parameter depends on the physical structure of the objects of interest as well as their textural characteristics. Selecting high scale parameters results in fewer, larger segments that can be bigger than the object observed, while lower scale parameter values result in smaller segments. However, the smaller the α, the longer the processing time (Table S2). The selection of the value of this parameter depends on the physical structure of objects as well as their textural characteristics; therefore, we selected the largest value that would provide efficient segmentation of each category of objects (HSCs, osteoblasts, and bone) throughout our datasets (Figure 2B).

To reconstruct the objects of interest from the segments obtained, we merged adjacent image segments based on the mean intensity difference to neighborhood (MDN) threshold. MDN threshold describes the difference between an image segment and its neighboring image segments, in terms of mean intensity values, and is defined as follows:

$$T_{\overline{\Delta_k}}(v) = \frac{1}{w} \sum_{u \in N_v(d)} w_u \left[\overline{c_k}(v) - \overline{c_k}(u) \right], \qquad \text{(Equation 1)}$$

where w is the image channel weight. Images are weighted by the distance between the segmented image objects, defined as follows:

$$w = \sum\nolimits_{u \in N_v(d)^{w_u}}, \qquad \text{(Equation 2)}$$

where v and u are two segmented image objects, N_V is the direct neighbors to the segmented image object v, u is defined as a direct neighbor to v if the minimum distance between them is less than or equal to d, d is the distance

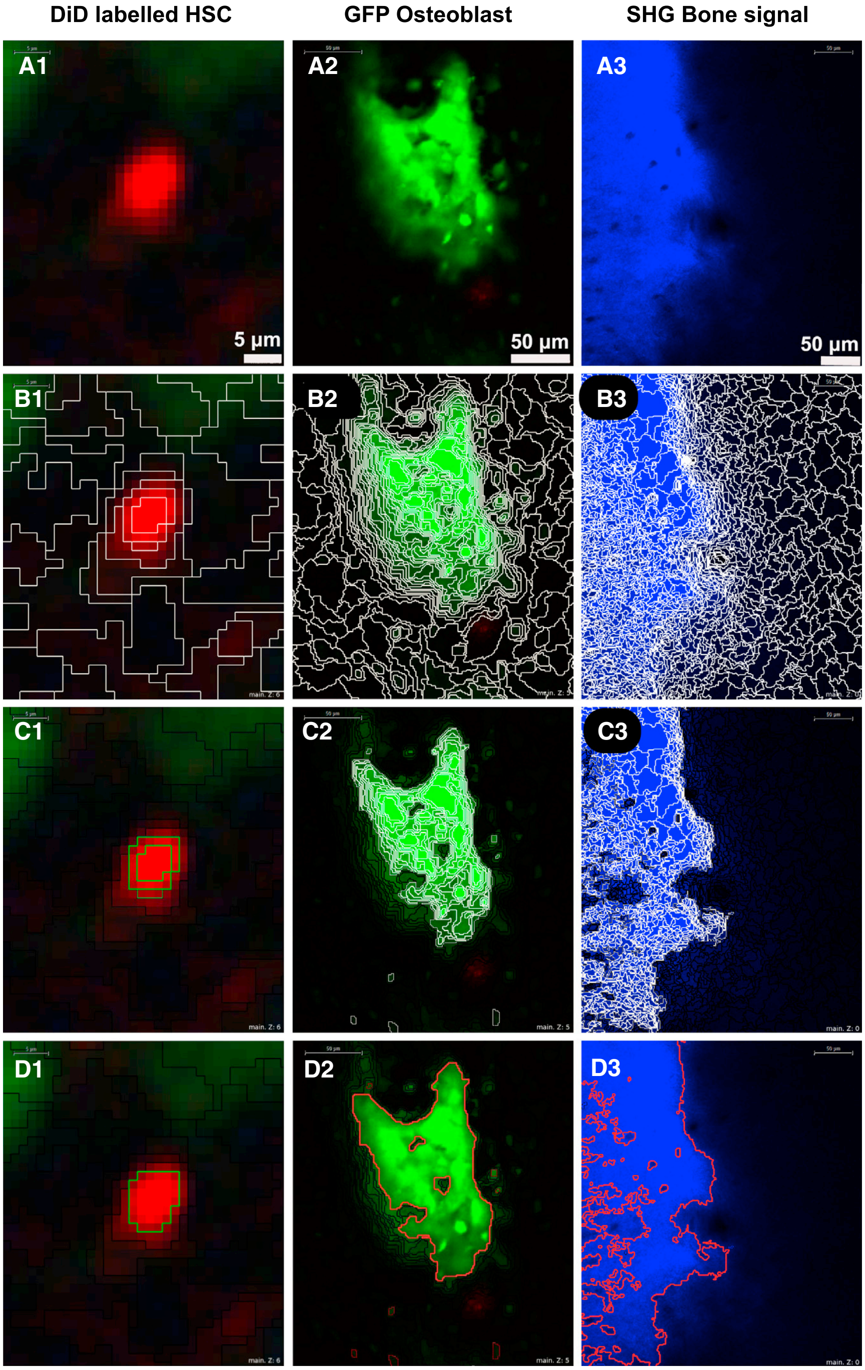

Figure 2. The LH-SEG Method

(A) Representative raw data from intravital microscopy experiments. (Left) Typical signal from a DiD-labeled HSC is shown. (Middle) GFP signal from osteoblast cells in a Col2.3GFP reporter mouse is shown. (Right) SHG signal obtained from 840-nm two-photon excitation of collagen to resolve bone structures is shown.

(B) Results of multi-resolution segmentation of each cellular component in (A) are shown.

(C) Results of MDN thresholding of each cellular component in (B) are shown.

(D) Results of automated detection of the components in (A) following segmentation and thresholding are shown.

between neighboring segments and defined as the radius of the segmented image object perimeter in pixel, w_u is the weight of the segmented image object defined by the difference of the mean intensity value between v and u in a given distance d, and $\overline{c_k}$ is the mean intensity value of channel k.

The appropriate MDN threshold for $T_{\overline{\Delta_k}}$ and distance feature d need to be selected for effective segmentation of each image object category (HSC, osteoblast, and bone). Progressive rounds of merges are repeated until the MDN of the resulting object is equal to or greater than a selected MDN threshold within a particular neighborhood distance. Given the different nature of HSCs, bone, and osteoblasts in terms of shape and intensity, we optimized different values for the MDN threshold and neighborhood distance parameters for each object category. DiD objects showed low-intensity heterogeneity and required a higher

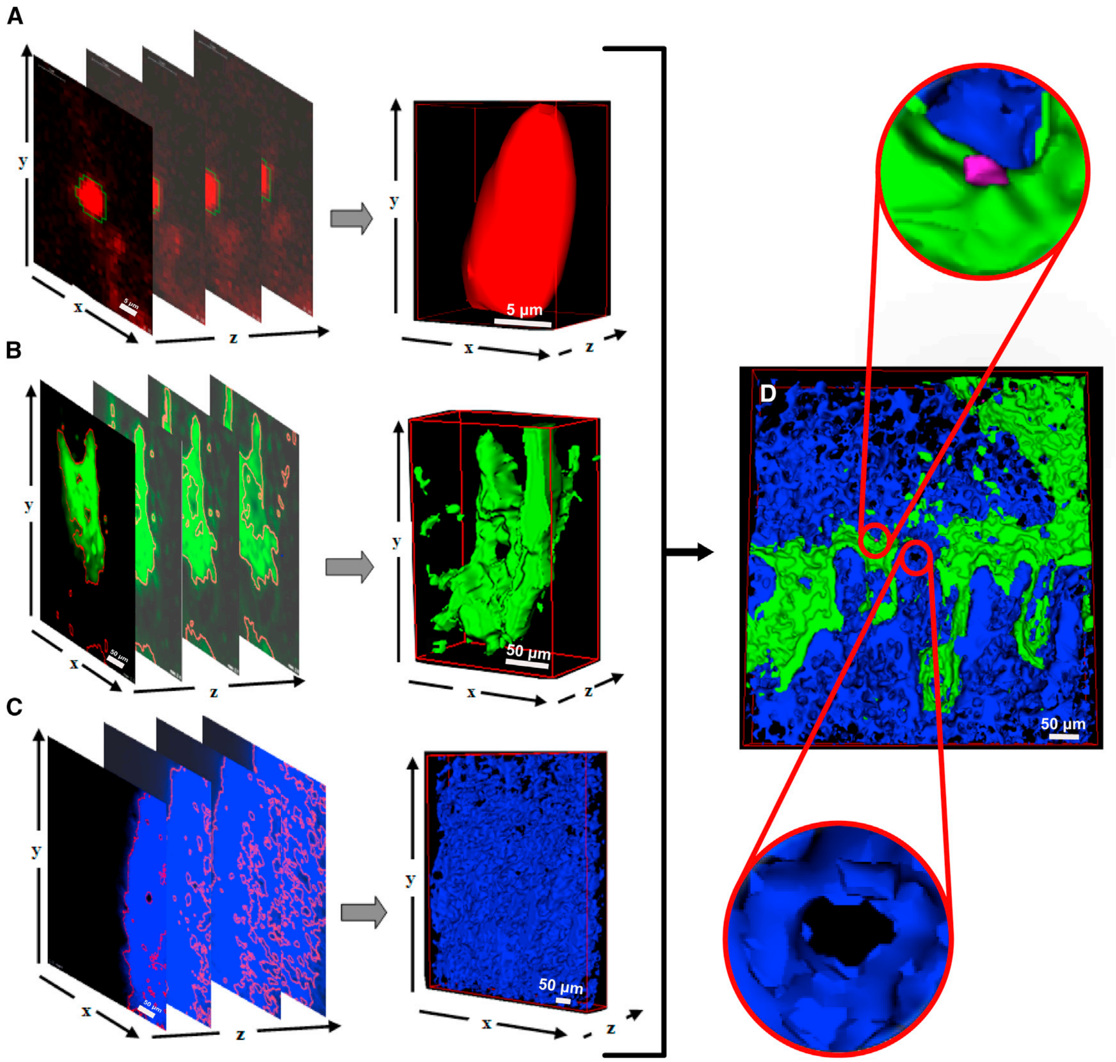

Figure 3. 3D Rendering of LH-Segmented 2D Slices

(A–C) Segmented objects in each 2D slice are merged across the z direction to form the 3D objects of each HSC niche component as follows: DiD-labeled HSCs/objects, red (A), GFP^+ osteoblastic cells, green (B), and SHG bone collagen signal, blue (C). (A)–(C) represent the 3D stacks of the 2D image shown in Figure 2A.

(D) 3D rendering of a complete three-channel stack. Insets show higher magnification images of a DiD-labeled HSC (magenta, top) and of a bone micro-cavity (bottom).

$T_{\overline{\Delta_k}}$ compared to bone and osteoblast clusters, which presented higher intensity heterogeneity. Conversely, the parameter distance d depends on the object size. DiD objects covered a smaller neighborhood compared to osteoblasts and bone, which are much larger; therefore, we selected a smaller d for DiD objects and a larger d for bone and osteoblasts (Table S3). The protocol for parameter selection and optimization for these and other types of objects is available in the Supplemental Experimental Procedures.

Once the objects were segmented in each 2D slice (Figures 2C and 2D), we linked them across the z dimension, according to the original z step size, to form the final 3D objects (Figure 3). Once the parameters were optimized on one initial dataset, we could apply them to the remaining nine datasets without any further alteration.

Machine Learning Classification of HSCs

A further challenge posed by the analysis of intravital microscopy images of chemically labeled HSCs is the discrimination between genuine HSCs and similar signal generated as a consequence of cell death and dye aggregation, or shedding of the dye onto neighboring cells and structures (Progatzky et al., 2013). While LH-SEG eliminated

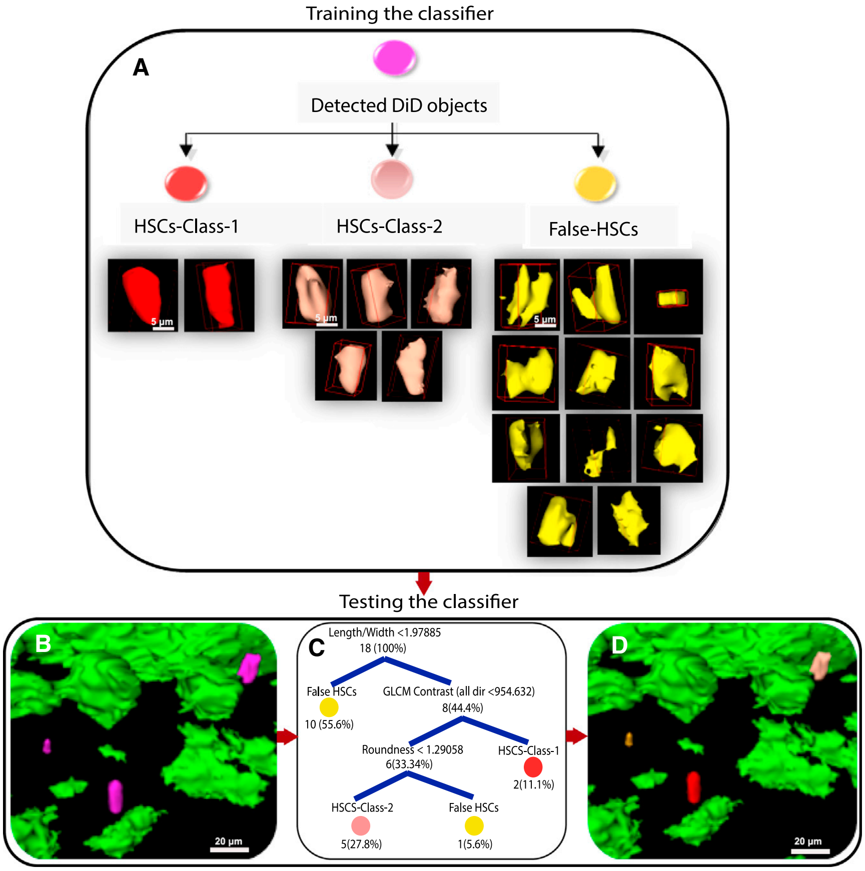

Figure 4. Machine Learning Training and Testing Using Decision Tree Classifier

(A) Training the classifier used the three classes of DiD objects as follows: HSCs-Class-1, where two representative HSC objects were used for training; HSCs-Class-2, where five representative HSC objects were used for training; and False-HSCs, where 11 objects representative of the spectrum of non-specific DiD signal shapes were used.

(B) Representative image of the 3D structure before machine learning classification is shown.

(C) The decision tree after training, representing the discriminative features selected by the classifier for each class based on the training set shown in (A) and indicating the discriminative threshold for each feature and the number of DiD objects after each ramification, is shown.

(D) Results of machine learning classification of the DiD-labeled objects in (B). Colors in (B)–(D) are coded according to (A).

the smallest DiD debris, aggregates and non-specifically labeled cells and structures remained in the segmented, 3D-reconstructed images and constituted false-positive signals that needed to be eliminated. As these objects share common characteristics with real HSCs, their elimination based solely on morphological and textural thresholds is a challenging task, and one often impossible to solve manually. To tackle this problem, we used a supervised 3D object-based classification approach to identify DiD signal as either positive (genuine) or negative (false) HSC objects. We selected the decision tree classifier (Agarwal and Sharma, 2011; Aydemir and Kayikcioglu, 2014) for this task due to its computational simplicity and illustrative attributes. The decision tree classifier weights boundaries between different classes based on their discriminative power. This type of classifier does not require a feature optimization task prior to classification; therefore, we could use a vast number of textural, intensity, and morphological features (Table S4) to train the classifier. It then automatically selected the discriminative features and the boundaries that defined different classes of DiD objects.

Due to the highly variable appearance of DiD objects, we manually prepared a training set containing three classes of DiD objects, based on the morphological characteristics (Figure 4). The first class, HSC-Class-1, comprises HSCs showing rounder shapes and smoother surfaces, as we would expect for quiescent, non-motile cells. The second class, HSC-Class-2, contains HSCs that have less rounded shapes and present small uropod protrusions (Krummel and Macara, 2006), as we previously observed in time-lapse images of migratory HSCs (Rashidi et al., 2014). The third class, False-HSCs, are DiD objects characterized by highly irregular morphology, for example, very pronounced protrusions, longer than the main diameter of the object itself, and objects that could represent cell doublets or clusters (Figure 4).

For the training task, two DiD objects were annotated as HSC-Class-1, five for HSC-Class-2, and 11 for False-HSCs (Figure 4). The number of samples was optimized for each class to avoid under-fitting and over-fitting the classifier. In fact, larger training samples resulted in increased false negatives. The size of each training set correlated with the heterogeneity of the objects within each class; thus, DiD objects that exhibited low variation, such as HSC-Class-1, required fewer samples compared to those with higher variation (HSC-Class-2 and False-HSCs).

To test whether machine learning could deal with our non-standardized datasets, all training samples were taken from one image dataset only (dataset 1, Table S1). We then tested the classifier on the nine remaining datasets. A 3-fold cross-validation was sufficient for training the classifier, as increasing the folds did not show any evidence of improving classification accuracy.

3D Localization: Distance Measurements

To minimize the complexity of the minimal distance search, we first extracted all the points that belonged to the surface of the HSC, osteoblast, and bone objects, and then we measured the distances between surface points of each object pair by computing their Euclidian distance (Ye, 1988), taking into account the anisotropic resolution of the image value, as follows:

$$\mathrm{dist} = \sqrt{(x1 - x2)^2 + (y1 - y2)^2 + [(z1 - z2)\cdot(sd)]^2}, \quad \text{(Equation 3)}$$

where dist is the distance between two points ($x1, y1, z1$ and $x2, y2, z2$), and sd is the distance between slices.

To calculate the minimum distance from each HSC to its neighboring osteoblast, we selected the closest point of the nearest osteoblast and the closest point of that particular HSC. Closest points of each pair were pseudo colored for visual identification of the nearest edges of associated objects. All analysis up to this point was performed using pixel units, and the results obtained here were then converted to micrometers (Figures 5A–5D). Of note, when we extracted positional data for control and infection-exposed HSCs, their distributions relative to osteoblasts were equivalent. However, when in a further experiment we injected, imaged, and analyzed myeloid progenitor (MP) cells (Lin^- $c\text{-}Kit^+$ $Sca\text{-}1^-$), they could be found further away from osteoblasts than HSCs. The position relative to endosteal surface was slightly but significantly different for HSCs and exp.HSCs (Figures 5E and 5F).

Performance Evaluation

Segmentation Accuracy

We evaluated the performance of the LH-SEG method by comparing its results to manually annotated data taken from each dataset. We also compared our proposed automated segmentation method to the following three commonly used segmentation approaches: automated local thresholding “Bernsen” (Sales et al., 2011), automated global thresholding (Otsu, 1979), and level-set segmentation, all available through the FIJI image adjustments and segmentation plugin (Schindelin et al., 2012). We evaluated each of these methods after optimization against the benchmarking manual segmentation and, therefore, could assess the improvement achieved by LH-SEG. We selected ten examples (one from each dataset) of DiD objects and GFP^+ osteoblasts that exhibited different intensity, edge morphology, and neighborhood characteristics. Bone signal was excluded from the quantitative segmentation evaluation, because the size of this structure makes it difficult to correctly select the bone region for the manual benchmarking. A user selected a 2D slice and segmented the objects manually (benchmarking); the manual segment was then converted into a mask and placed over the automated segmentation mask for comparison. Regions of the automated segmentation that matched the manual segmentation regions were considered true positives (*TP*), regions of the automated segmentation that did not match with the manual segmentation regions were false positives (*FP*), and regions of the manual segmentation missed by the automated segmentations were false negatives (*FN*)(Figures S1 and S2).

In the case of the DiD objects (Figure S1), automated local thresholding parameters were optimized for the first image and provided good results; however, when the same parameters were used to segment DiD objects from other images, they led to poor segmentation. Thus, this method would require optimization for each individual image and would only give high *TP* values for DiD objects that exhibit high-intensity levels and result in high *FN* values when DiD objects exhibited medium to low intensities.

Global thresholding yielded poor results and was difficult to optimize due to the nature of DiD signal, which exhibits low contrast with the background and a high level of noise.

Level-set segmentation provided high *TP* in cases where HSCs exhibited high contrast between the cell edges and the background and increased *FP* in cases where a high level of background noise around the cell edges was found. Our proposed LH-SEG method maintained consistency in segmenting HSCs regardless of the DiD objects intensity, contrast level, and background noise and provided high *TP* and low *FN* and *FP* in all tested data using only one set of optimized parameters.

Automated local thresholding and level-set segmentation yielded poor results for osteoblasts, which exhibit high-intensity heterogeneity (Figure S2). Both methods were only able to detect osteoblastic regions that exhibited high-intensity levels compared to their surrounding neighborhood, making it impossible to optimize the parameters to work across all our image datasets. Osteoblastic regions of intermediate intensity were not detected using these methods and resulted in increased *FP*. Global thresholding resulted in increased *FP* when images included osteblastic regions of heterogeneous intensities. However, when osteoblasts had homogeneous intensity and good contrast

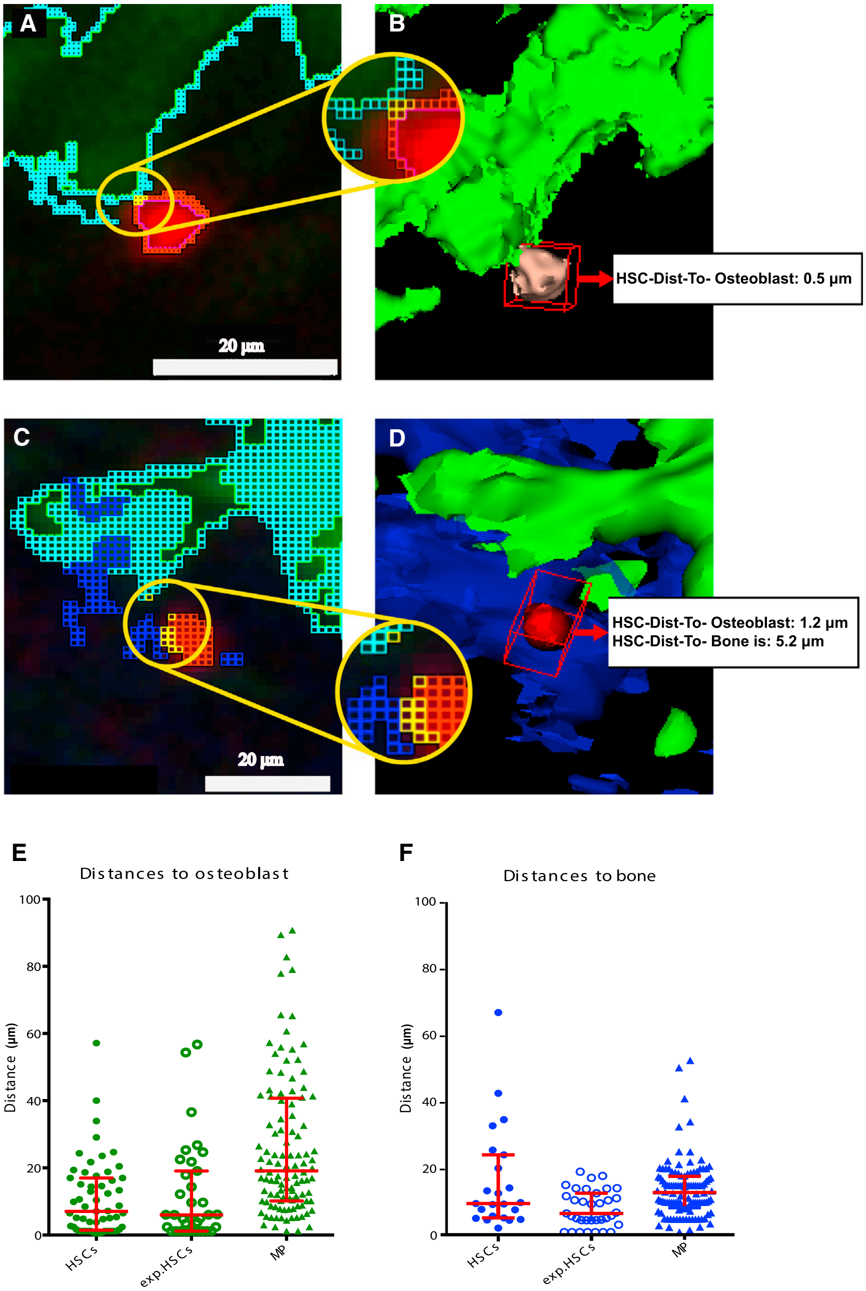

Figure 5. Nearest Edge Detection and HSC-to-Osteoblast/Bone Signal 3D Shortest Distance Measurements

(A) HSC and osteoblastic cells surface detection and nearest edge localization. Red pixels represent HSC surface, cyan pixels represent osteoblast surface, and yellow pixels represent the nearest edges of each pair (HSC and osteoblast).

(B) 3D distance measurement from selected HSC to nearest osteoblast is shown.

(C) HSC, osteoblastic cells, and bone surface detection and nearest edge localization. In addition to colors as in (A), blue pixels represent bone surface.

(D) 3D distance measurement from selected HSC to nearest osteoblast and bone. Examples of shortest distances that happened to be within the same 2D slice were selected for simplicity.

(E and F) Distance measurements from HSCs, infection-exposed HSCs (exp.HSCs), and MPs to the nearest osteoblast (E) and bone signal (F). P values (Mann-Whitney U test for non-Gaussian parameter distribution) are 0.9503 (HSCs versus exp.HSCs, Ob), 0.0383 (HSCs versus exp.HSCs, bone), <0.0001 (HSCs versus MPs, Ob), and 0.5115 (HSCs versus MPs, bone). n = 57 and 23 HSCs measured to osteoblasts and bone, respectively, 35 exp.HSCs, 112 MPs from ten independent experiments (datasets 1, 3, 5–10, Table S1, and two large area tiles). Error bars, median and interquartile range.

compared to the background, global thresholding provided high *TP* and low *FN* and *FP*. Our proposed LH-SEG method maintained consistency in segmenting osteoblastic cell regions regardless of their intensity, contrast level, and background noise and provided high *TP* and low *FN* and *FP* across all ten tested datasets, despite using only one single set of optimized parameters.

To measure the accuracy of each automated segmentation method, we used the Jaccard similarity index defined as follows:

$$J(M,A)=\frac{|M\cap A|}{|M\cup A|}, \qquad \text{(Equation 4)}$$

where A represents the automated segmentation results and M is the manual benchmarking data, $M\cap A$ is the *TP* regions, and $M\cup A$ is the sum of *TP*, *FP*, and *FN* regions. LH-SEG achieved the highest and most reproducible J values for both HSC and osteoblast segmentation (Figure 6A). Qualitative evaluation of LH-SEG of bone showed no evidence of it being

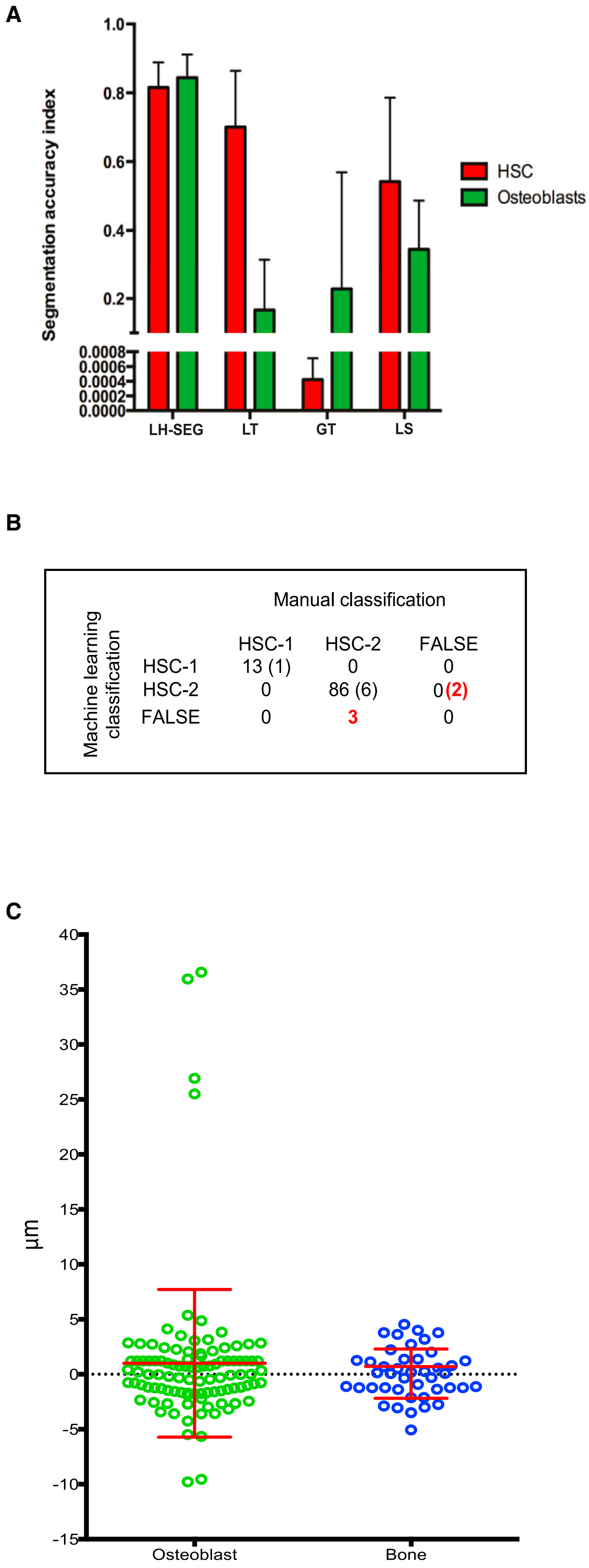

Figure 6. Evaluation of LH-SEG Segmentation, Classification, and 3D Distance Measurement Accuracy for All Ten Datasets

(A) Evaluation of LH-SEG segmentation accuracy using the Jaccard similarity index. LH-SEG segmentation outperformed the other methods considered and achieved an accuracy index average of 0.82 for bone and osteoblasts. All other segmentation methods achieved lower scores. LT, local thresholding; GT, global thresholding; LS, level-set segmentation. n = 10 independent regions, one from each dataset. Error bars, mean ± SD.

(B) Manual benchmark and machine learning classification. Columns indicate the results of manual (benchmark) classification and rows indicate the classification results obtained with machine learning. Red highlights indicate machine learning classification error, and in parentheses are objects that were identified and classified by machine learning but that had been missed manually.

(C) Evaluation of automated 3D distance measurement by means of measured errors. Green circles represent the difference between manual and automated distance measurements from HSCs to osteoblast. Blue circles represent the difference between manual and automated distance measurements from HSCs to bone. Each circle is from a single measured HSC. Error bars, mean ± SD; n = 102 HSC-to-Ob measurements from the ten independent datasets listed in Table S1 and 43 HSC-to-bone measurements from datasets 8–10 (Table S1).

any less accurate than that of HSCs and osteoblasts (Figure 2).

Machine Learning Classification Accuracy

Manual classification (benchmark) was performed on unsegmented images and included three classes of objects as follows: HSC-Class-1, HSC-Class-2, and False-HSCs, as we used to train the classifier. We did not manually classify all False-HSCs as it was unfeasible due to their large number; therefore, any DiD object that was not classified in any of the three manual classes was automatically considered a benchmark False-HSC.

The classifier assessed all DiD objects present in the ten datasets (Table S1) and classified them as HSC-Class-1, HSC-Class-2, and False-HSCs according to the training set. This identified 14 HSC-Class-1 and 94 HSC-Class-2, including seven bona fide HSCs that were manually missed, however, correctly identified and classified by the classifier. All remaining signal was classified as False-HSCs. This was consistent with the fact that only few HSCs are observed in each imaged mouse (i.e., in each dataset) (Lo Celso et al., 2011). We then evaluated the classification results by calculating the Precision and Recall values defined in Equations 5 and 6.

$$\text{Precision} = \frac{TP}{TP + FP} \qquad \text{(Equation 5)}$$

$$\text{Recall} = \frac{TP}{TP + FN} \qquad \text{(Equation 6)}$$

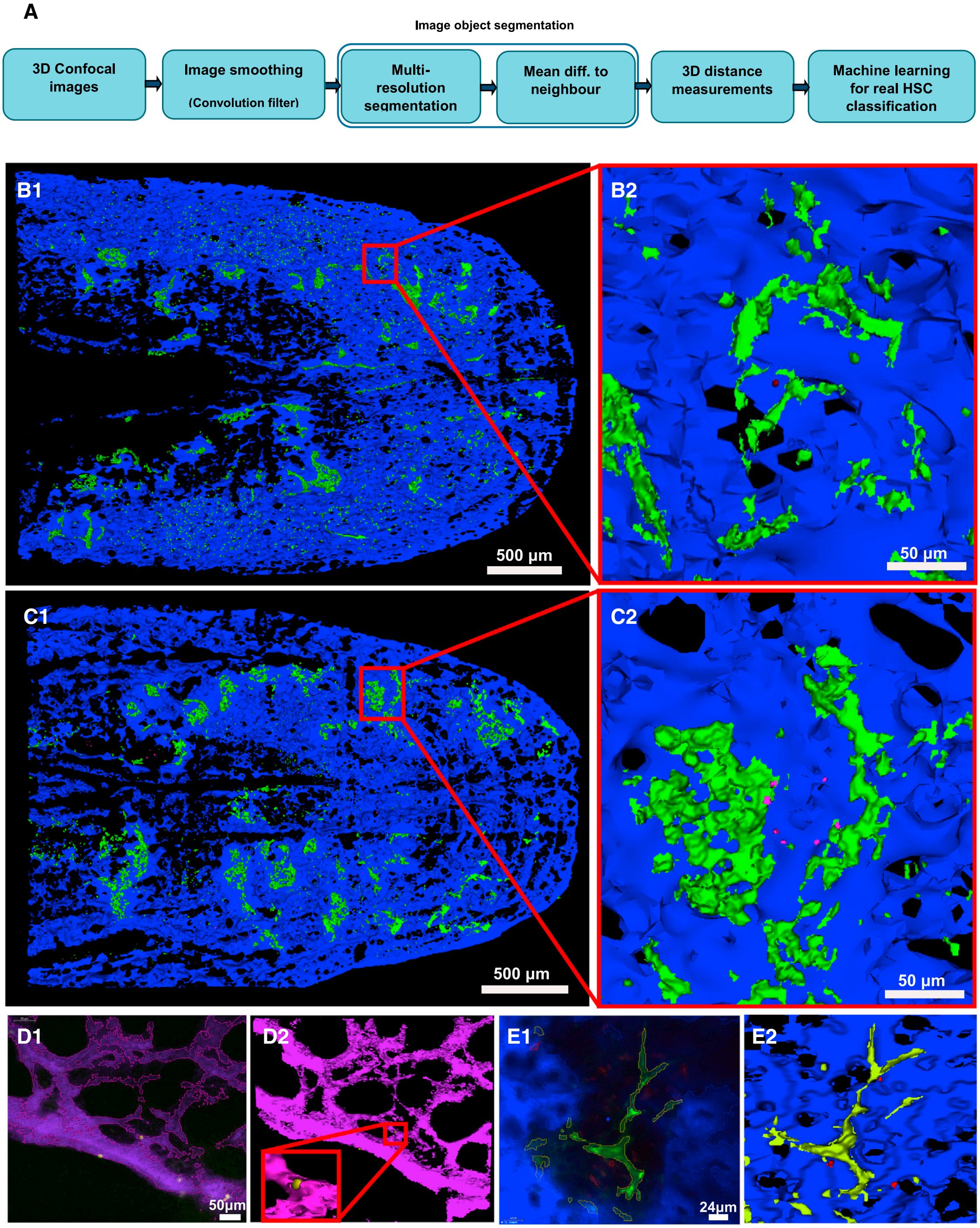

Figure 7. Overview of Our Automated Pipeline and Its Applicability to Large Areas of Bone Marrow and a Wide Range of Niche Components

(A) Raw data are subjected to an initial pre-processing smoothing. LH-SEG identifies DiD-labeled objects, osteoblastic cells, and bone collagen; the 2D objects resulting from the segmentation are linked in 3D according to the raw data step size, then the decision tree classifier separates HSCs from DiD non-specific signal and HSC-to-osteoblast and HSC-to-bone shortest 3D distances are measured.

(B) (Left) 3D rendering of a calvarium tile scan including DiD-labeled HSC signal in red, GFP+ osteoblasts in green, and SHG bone collagen in blue. (Right) Higher magnification image of a detail of the tile scan is shown.

(legend continued on next page)

where TP represents the number of correctly classified bona fide HSCs, FP represents the number of false HSCs classified as bona fide HSCs, and FN represents the number of bona fide HSCs classified as false HSCs. The Precision value represents the fraction of correctly classified bona fide HSCs, while the Recall value represents the fraction of HSCs selected by the classifier that are bona fide HSCs. Precision and Recall are = 1.0 if the classifier does not report any errors. Based on all tested 102 bona fide HSCs, only three bona fide HSCs were misclassified by machine learning and were FN and two classified HSCs were FP (Figure 6B), giving a Precision value of 0.98 and a Recall value of 0.97.

3D Localization Accuracy

Although segmentation accuracy plays a vital part in determining the efficacy of the minimum distance measurements, it is also important to evaluate the performance of the automated distance measurement algorithm. Distance measurement benchmarking was done manually (Lo Celso et al., 2009) using the commercial software Volocity, with users drawing lines between two points manually selected to be the closest edges of the selected DiD object and neighboring osteoblast/bone within unsegmented 3D images. Manual 3D distance measurements were set to 0 when DiD-labeled objects touched osteoblast or bone. All manual and automated measures are presented in Figure S3.

To evaluate the results of the automated 3D distance measurements, we calculated the error percentage for all ten datasets combined as defined in Equation 7.

$$\% \text{ Error} = \left| \frac{\bar{d}(M) - \bar{d}(A)}{\bar{d}(M)} \right| \cdot 100, \qquad \text{(Equation 7)}$$

where $\bar{d}(M)$ is the number of DiD objects used for the manual 3D distance measurement benchmarking, and $\bar{d}(A)$ is the number of DiD objects where the automated 3D distance measurement matched the benchmarking measurements. Distance measurements $\overline{d_m}$ and $\overline{d_a}$ (Equation 8) were a match M if their difference was ≤ 5 μm, which is the upper margin of the error expected when measuring 3D distances manually (Figure 6C).

$$\left|\overline{d_m} - \overline{d_a}\right| \leq 5\mu\text{m} \cdot |M|, \qquad \text{(Equation 8)}$$

where $\overline{d_m}$ is the manual distance measurement of HSCs to nearest osteoblast/bone, and $\overline{d_a}$ is the automated one.

Automated HSC to osteoblast localization measurements proved to be accurate when compared to the manual 3D distance measurements (with 6.8% error based on the 102 HSCs measured). Furthermore, automated HSC-to-bone localization measurements resulted in no error based on all 43 HSCs in the datasets that had bone signal.

Wide Applicability of LH-SEG and 3D Measurements

Given the size and complexity of bone marrow tissue, we examined whether our method would allow analysis of areas larger than a single field of view and of hematopoietic cells and niche components other than DiD-labeled HSCs, osteoblasts, and bone (Figure 7A). We injected DiD-labeled HSCs in a Col2.3GFP recipient, and, instead of searching for HSCs and acquiring fields of view that contained promising DiD signal, we set up a tiled acquisition of a calvarium area of approximately 4,413 × 3,272 × 125 μm. Our method successfully segmented osteoblasts, bone, and DiD signal; classified DiD signal; and identified six bona fide HSCs in locations equivalent to those normally acquired as single fields of view (Figure 7B). Also, using the same approach, we were able to identify and measure tomato+ committed MPs (Figures 7C, 5E, and 5F). Machine learning classification was not applied to this type of cell, as it is only needed to distinguish chemically labeled cells from cell debris and aggregates.

Next we tested whether other bone marrow components would be analyzable using our method. We imaged wild-type recipients reconstituted with MacBlue bone marrow, in which cells of the monocytic-macrophage lineage express CFP (Hume, 2011), and injected with TRITC-labeled dextran to highlight all vasculature immediately prior to imaging. By identifying appropriate LH-SEG parameters for macrophages and vessels, we were able to segment and 3D render macrophages adjacent to bone marrow sinusoids (Figure 7D). Similarly, we identified tomato-expressing MPs injected in a nestin GFP recipient (Méndez-Ferrer et al., 2010) and measured their position relative to GFP-expressing nestin+ mesenchymal progenitors (Figure 7E; data not shown).

(C) (Left) 3D rendering of a calvarium tile scan including tomato$^+$ progenitor cells in magenta, GFP$^+$ osteoblasts in green, and SHG bone collagen in blue. (Right) Higher magnification image of a detail of the tile scan is shown.
(D) (Left) Representative example of automated detection of TRITC dextran-labeled blood vessels (purple) and CFP+ macrophages (yellow) following LH-SEG segmentation on a 2D slice. (Right) 3D rendering after segmentation and linking of each 2D slice in the z stack containing the image shown on the left. Inset shows higher magnification of a detail of the 3D rendering.
(E) (Left) Representative example of automated detection of nestin cells (green), tomato$^+$ progenitors (red), and SHG bone signal (blue) following LH-SEG segmentation on a 2D slice. (Right) 3D rendering after segmentation and linking of each 2D slice in the z stack containing the image shown on the left.

DISCUSSION

We have developed an automated segmentation, classification, and localization measurement algorithm (Figure 7A) for analysis of confocal and two-photon microscopy images of HSCs, osteoblasts, and bone obtained through intravital microscopy of mouse bone marrow. In particular, we addressed the following two main challenges posed by HSC intravital microscopy image analysis: (1) non-standardized datasets, which vary based on each specific experiment and user; and (2) highly variable image quality and signal intensity, due to the complexity of the structures observed and the increasing signal loss and scatter with depth of imaging. The use of non-standardized datasets with varying acquisition settings (i.e., magnification, excitation power, detector gain, and z step size), reflecting the non-standardized nature of intravital microscopy experiments, allowed us to test and demonstrate the robustness of the proposed approach.

While the majority of segmentation algorithms work best with objects exhibiting a narrow range of signal intensity, LH-SEG could deal with highly heterogeneous objects because it recognizes the intensity differences between each object and its neighborhood, independently of the size of the object. This method, therefore, did not require any assumption on the size or shape of the objects segmented, and was particularly suited to segment bone and osteoblast clusters as they exhibit variable morphology.

Furthermore, machine learning classification of DiD objects separated genuine HSCs from DiD debris and was sufficiently flexible to recognize the variable appearance of HSCs. In our experience, this is the most subjective step of manual analysis of HSC images, leading to some HSCs not being included in the analysis (false negative) and some debris being further analyzed instead. The decision tree machine learning approach, which automates the feature optimization task, efficiently selected the few discriminative features that best identified HSCs from false signal. Of note, this method proved slightly more effective than manual analysis of 3D stacks, as it allowed detection and analysis of a higher number of HSCs (seven cells had not been recognized by the human eye). Most importantly, it sped up the analysis process and increased its objectivity, therefore improving current manual analysis. Of note, 3D stacks are acquired by definition in areas where the user has identified HSCs being present, and, therefore, the margin left for detection improvement is small. Our analysis method is critically helpful when large, non-pre-selected areas of bone marrow are acquired and analyzed, such as the 3D tiles we tested, which are extremely time consuming to be analyzed manually.

Our analysis algorithm segments, classifies, and localizes HSCs in their natural niche based on their morphology, texture, and surroundings, modeling the processes that the human brain uses to identify and understand relational features of the HSCs within an image. As a result, we obtained a high level of accuracy for segmentation, classification, and localization measurements, and only few errors, discussed in detail below, were reported for the classification of DiD objects (5/111 total cells identified by machine learning or manual user) and HSC localization (7/102 HSC-to-osteoblast and 0/43 HSC-to-bone measures). Classification errors occurred between HSC-Class-2 and False-HSCs, likely due to the common characteristics that both types of DiD objects share (less rounded shapes and uropod/irregular protrusions), which can mislead the classifier. Given their low frequency, these errors are unlikely to affect further analysis and, therefore, do not impinge on the validity of the automated results obtained.

Localization error was reported in circumstances where the automated approach measured the distance to a brighter osteoblast while a dimmer osteoblast was detected manually (2/102), or vice versa (2/102). In both cases, this type of error could be further rectified by increasing or decreasing the MDN threshold for the specific 3D stack; however, the resulting values may not be applicable to the majority of the other datasets. It was more efficient to have values that yielded rare errors across highly variable datasets than to have to manually select new values for each dataset generated. Another source of localization error was the position of HSC in the 3D stack (3/102): HSCs positioned at the edge of the xy field may be closer to osteoblasts located outside the field of view, and HSCs positioned too deep in the bone marrow yield unreliable measures because the signal is too distorted (and they could be closer to osteoblasts/endosteal surface on the cavity bottom [Lo Celso et al., 2009]). These HSCs are easily identifiable when collecting the data and simply should be eliminated from the analysis, as even manually it would be impossible to provide a reliable measurement.

Of note, the median of the differences between all manual and automated distance measurements was zero, indicating that the error in our method is unbiased relative to manual measurements and, therefore, our approach is not skewing the results obtained. Thus, the overall distribution of HSC positions observed with our automated algorithm was equivalent to that obtained manually from previously published datasets (Lane et al., 2011; Lo Celso et al., 2009), with the advantage that they were obtained in a fraction of the time.

As little is known about the spatial organization of hematopoietic and stroma cells in the bone marrow, our method will be useful to further elucidate the relative position of multiple cell types. Here we identified that

exp.HSCs, despite being motile, are found to have the same positional distribution relative to osteoblasts as steady-state HSCs, while MP distribution is wider. The observed differing distributions relative to osteoblasts and endosteum may reflect biological differences.

Our image analysis tool detects and analyzes single DiD-labeled HSCs. Methods to distinguish doublets, clusters, and dividing cells will require further refinement. However, the workflow described here allows automated segmentation, classification, and localization of HSCs during the initial stage of engraftment (homing), a critical step for the success of bone marrow transplantation therapeutic protocols, and one for which evidence has been accumulated of the correlation between HSC position relative to niche cells/components and their long-term function (Lo Celso et al., 2009; Lane et al., 2011). HSC proliferation may be better studied using different experimental approaches altogether, based on endogenous expression of fluorescent reporters rather than chemical dyes that dilute upon cell division (Progatzky et al., 2013).

Our approach was developed to detect and analyze DiD-labeled HSCs, osteoblasts, and bone collagen, but it was immediately expandable to the analysis of further bone marrow niche components, including ones with highly variable appearance, such as nestin cells and blood vessels. While analysis of large areas of bone marrow did not increase the number of HSCs detected per mouse, this approach considerably simplified and sped up acquisition and analysis of images, especially those with large numbers of cells, such as MPs. Based on our data, we can conclude that our approach is applicable to the analysis of a broad range of 3D and intravital microscopy images from other tissues and organs, leading to a faster pace of discovery.

EXPERIMENTAL PROCEDURES

Generation of Raw Data by Intravital Microscopy

All animal work was performed according to the UK Home Office Animals (Scientific Procedures) Act regulations and was approved by the Imperial College Ethics committee and by the Home Office. Intravital microscopy of HSCs was performed as described previously (Lo Celso et al., 2009; Rashidi et al., 2014), with the donor and recipient mice listed in Table S1. The lipophilic dye DiD was used to label all HSCs (LKS CD150^{+} CD48^{-} or LKS CD34^{-} Flk2^{-}). In some experiments, Lin^{-} c-Kit^{+} Sca-1^{-} MPs were purified from the bone marrow of mT/mG donor mice (Muzumdar et al., 2007), injected into irradiated Col2.3GFP osteoblast (Kalajzic et al., 2002) and Nestin-GFP (Méndez-Ferrer et al., 2010) reporter mice, and identified as tomato+ cells. MacBlue bone marrow was a kind gift of Professor D. Hume (Edinburgh University) and was injected into irradiated wild-type recipients. Once fully reconstituted, mice were injected with 50 μl of 40 mg/ml TRITC-dextran to label blood vessels. All imaged mice were anesthetized and their scalps were removed and replaced with imaging windows (Rashidi et al., 2014). All microscopy was performed at Imperial College with a Leica SP5 (Facility for Imaging by Light Microscopy) and a Zeiss LSM 780 upright confocal/two-photon combined microscopes (Scott et al., 2014).

Automated Method Implementation and Availability

The presented method was developed in Definiens Developer XD 64. The source code is available in the Supplemental Information available online (Segment-Classify-Measure-distance-3D.dcp).

Computations were conducted on Intel-Core i5-3427U processor at 1.80 GHz and 4.0 GB RAM, running the 64-bit Windows 7 operating system. The total processing time per 3D stack is 5 min 26 s for a typical 512 × 512 × 18 (x,y,z)-pixel image size with three channels for DiD signal, GFP^{+} osteoblast cells, and SHG bone collagen signal, and where two real HSCs were identified. Analysis of large datasets can be carried out without manual supervision. The processing time varies depending on the 3D stack size/depth and the number of HSCs found.

SUPPLEMENTAL INFORMATION

Supplemental Information includes Supplemental Experimental Procedures, three figures, four tables, and one movie and can be found with this article online at http://dx.doi.org/10.1016/j.stemcr.2015.05.017.

ACKNOWLEDGMENTS

The Kay Kendall Leukaemia Fund, Human Frontiers Science Program, Cancer Research UK, European Research Council, and Biotechnology and Biological Sciences Research Council supported this work. E.D.H. was supported by a European Hematology Association Fellowship. We thank Dr. D. Keller (Imperial College Facility for Imaging by Light Microscopy) for technical support; Professor D. Hume and Dr. C. Pridans for kindly providing MacBlue bone marrow; and Professor K. Duffy, Dr. L. Pizarro, and Dr. A. Dufour for comments on the manuscript.

Received: December 12, 2014
Revised: May 29, 2015
Accepted: May 29, 2015
Published: June 25, 2015

REFERENCES

Agarwal, C., and Sharma, A. (2011). Image understanding using decision tree based machine learning. Proceedings of the 2011 International Conference on Information Technology and Multimedia (ICIM), 1–8.

Aydemir, O., and Kayikcioglu, T. (2014). Decision tree structure based classification of EEG signals recorded during two dimensional cursor movement imagery. J. Neurosci. Methods *229*, 68–75.

Baatz, M., and Schäpe, A. (2000). Multiresolution segmentation-an optimization approach for high quality multi-scale image segmentation. In Angew. Geogr. Info. verarbeitung, J. Strobl, T. Blaschke, and G. Griesebner, eds. (Heidelberg: Wichmann-Verlag), pp. 12–23.

Calvi, L.M., Adams, G.B., Weibrecht, K.W., Weber, J.M., Olson, D.P., Knight, M.C., Martin, R.P., Schipani, E., Divieti, P., Bringhurst, F.R., et al. (2003). Osteoblastic cells regulate the haematopoietic stem cell niche. Nature *425*, 841–846.

Chung, G., and Vese, L.A. (2009). Image segmentation using a multilayer level-set approach. Comput. Vis. Sci. *12*, 267–285.

Fleming, H.E., Janzen, V., Lo Celso, C., Guo, J., Leahy, K.M., Kronenberg, H.M., and Scadden, D.T. (2008). Wnt signaling in the niche enforces hematopoietic stem cell quiescence and is necessary to preserve self-renewal in vivo. Cell Stem Cell *2*, 274–283.

Hume, D.A. (2011). Applications of myeloid-specific promoters in transgenic mice support in vivo imaging and functional genomics but do not support the concept of distinct macrophage and dendritic cell lineages or roles in immunity. J. Leukoc. Biol. *89*, 525–538.

Kalajzic, Z., Liu, P., Kalajzic, I., Du, Z., Braut, A., Mina, M., Canalis, E., and Rowe, D.W. (2002). Directing the expression of a green fluorescent protein transgene in differentiated osteoblasts: comparison between rat type I collagen and rat osteocalcin promoters. Bone *31*, 654–660.

Köhler, A., Schmithorst, V., Filippi, M.D., Ryan, M.A., Daria, D., Gunzer, M., and Geiger, H. (2009). Altered cellular dynamics and endosteal location of aged early hematopoietic progenitor cells revealed by time-lapse intravital imaging in long bones. Blood *114*, 290–298.

Krummel, M.F., and Macara, I. (2006). Maintenance and modulation of T cell polarity. Nat. Immunol. *7*, 1143–1149.

Kunisaki, Y., Bruns, I., Scheiermann, C., Ahmed, J., Pinho, S., Zhang, D., Mizoguchi, T., Wei, Q., Lucas, D., Ito, K., et al. (2013). Arteriolar niches maintain haematopoietic stem cell quiescence. Nature *502*, 637–643.

Lane, S.W., Wang, Y.J., Lo Celso, C., Ragu, C., Bullinger, L., Sykes, S.M., Ferraro, F., Shterental, S., Lin, C.P., Gilliland, D.G., et al. (2011). Differential niche and Wnt requirements during acute myeloid leukemia progression. Blood *118*, 2849–2856.

Lo Celso, C., Fleming, H.E., Wu, J.W., Zhao, C.X., Miake-Lye, S., Fujisaki, J., Côté, D., Rowe, D.W., Lin, C.P., and Scadden, D.T. (2009). Live-animal tracking of individual haematopoietic stem/progenitor cells in their niche. Nature *457*, 92–96.

Lo Celso, C., Lin, C.P., and Scadden, D.T. (2011). In vivo imaging of transplanted hematopoietic stem and progenitor cells in mouse calvarium bone marrow. Nat. Protoc. *6*, 1–14.

Lou, X., Kang, M., Xenopoulos, P., Muñoz-Descalzo, S., and Hadjantonakis, A.K. (2014). A rapid and efficient 2D/3D nuclear segmentation method for analysis of early mouse embryo and stem cell image data. Stem Cell Reports *2*, 382–397.

Mallinis, G., Koutsias, N., Tsakiri-Strati, M., and Karteris, M. (2008). Object-based classification using Quickbird imagery for delineating forest vegetation polygons in a Mediterranean test site. ISPRS J. Photogramm. Remote Sens. *63*, 237–250.

Méndez-Ferrer, S., Michurina, T.V., Ferraro, F., Mazloom, A.R., Macarthur, B.D., Lira, S.A., Scadden, D.T., Ma'ayan, A., Enikolopov, G.N., and Frenette, P.S. (2010). Mesenchymal and haematopoietic stem cells form a unique bone marrow niche. Nature *466*, 829–834.

Moon, B.S. (2001). A Gaussian smoothing algorithm to generate trend curves. Korean J. Comput. Appl. Math. *8*, 507–518.

Morrison, S.J., and Scadden, D.T. (2014). The bone marrow niche for haematopoietic stem cells. Nature *505*, 327–334.

Muzumdar, M.D., Tasic, B., Miyamichi, K., Li, L., and Luo, L. (2007). A global double-fluorescent Cre reporter mouse. Genesis *45*, 593–605.

Nandy, K., Kim, J., McCullough, D.P., McAuliffe, M., Meaburn, K.J., Yamaguchi, T.P., Gudla, P.R., and Lockett, S.J. (2014). Segmentation and quantitative analysis of individual cells in developmental tissues. Methods Mol. Biol. *1092*, 235–253.

Otsu, N. (1979). A threshold selection method from gray-level histograms. IEEE Trans. Syst. Man Cybern. *9*, 62–66.

Pop, S., Dufour, A.C., Le Garrec, J.F., Ragni, C.V., Cimper, C., Meilhac, S.M., and Olivo-Marin, J.C. (2013). Extracting 3D cell parameters from dense tissue environments: application to the development of the mouse heart. Bioinformatics *29*, 772–779.

Progatzky, F., Dallman, M.J., and Lo Celso, C. (2013). From seeing to believing: labelling strategies for in vivo cell-tracking experiments. Interface Focus *3*, 20130001.

Rashidi, N.M., Scott, M.K., Scherf, N., Krinner, A., Kalchschmidt, J.S., Gounaris, K., Selkirk, M.E., Roeder, I., and Lo Celso, C. (2014). In vivo time-lapse imaging shows diverse niche engagement by quiescent and naturally activated hematopoietic stem cells. Blood *124*, 79–83.

Ritsma, L., Ellenbroek, S.I., Zomer, A., Snippert, H.J., de Sauvage, F.J., Simons, B.D., Clevers, H., and van Rheenen, J. (2014). Intestinal crypt homeostasis revealed at single-stem-cell level by in vivo live imaging. Nature *507*, 362–365.

Rompolas, P., Deschene, E.R., Zito, G., Gonzalez, D.G., Saotome, I., Haberman, A.M., and Greco, V. (2012). Live imaging of stem cell and progeny behaviour in physiological hair-follicle regeneration. Nature *487*, 496–499.

Saikumar, T., Nagarani, M., Yojana, K., and Shashidhar, B. (2012). Image segmentation of an adaptive threshold algorithm using watershed transform and fuzzy c-means clustering on level set method. Proceedings of the 2012 International Conference on Advances in Engineering, Science and Management (ICAESM), 33–38.

Sales, E., Gomez, W., and Pereira, W.C.A. (2011). Evaluation performance of local adaptive binarization algorithms for trabecular bone on simulated μCT. Proceedings of the 2011 IEEE Nuclear Science Symposium and Medical Imaging Conference (NSS/MIC), 3084–3087.

Sanchez-Aguilera, A., Lee, Y.J., Lo Celso, C., Ferraro, F., Brumme, K., Mondal, S., Kim, C., Dorrance, A., Luo, H.R., Scadden, D.T., and Williams, D.A. (2011). Guanine nucleotide exchange factor Vav1 regulates perivascular homing and bone marrow retention of hematopoietic stem and progenitor cells. Proc. Natl. Acad. Sci. USA *108*, 9607–9612.

Scadden, D.T. (2014). Nice neighborhood: emerging concepts of the stem cell niche. Cell *157*, 41–50.

Schindelin, J., Arganda-Carreras, I., Frise, E., Kaynig, V., Longair, M., Pietzsch, T., Preibisch, S., Rueden, C., Saalfeld, S., Schmid, B., et al. (2012). Fiji: an open-source platform for biological-image analysis. Nat. Methods *9*, 676–682.

Scott, M.K., Akinduro, O., and Lo Celso, C. (2014). In vivo 4-dimensional tracking of hematopoietic stem and progenitor cells in adult mouse calvarial bone marrow. J. Vis. Exp. *91*, e51683.

Ye, Q.-Z. (1988). The signed Euclidean distance transform and its applications. Proceedings of the 9th International Conference on Pattern Recognition, 495–499.

Yeo, S.Y., Xie, X., Sazonov, I., and Nithiarasu, P. (2011). Level set segmentation with robust image gradient energy and statistical shape prior. Proceedings of the 2011 18th IEEE International Conference on Image Processing (ICIP), 3397–3400.

Figure S1.

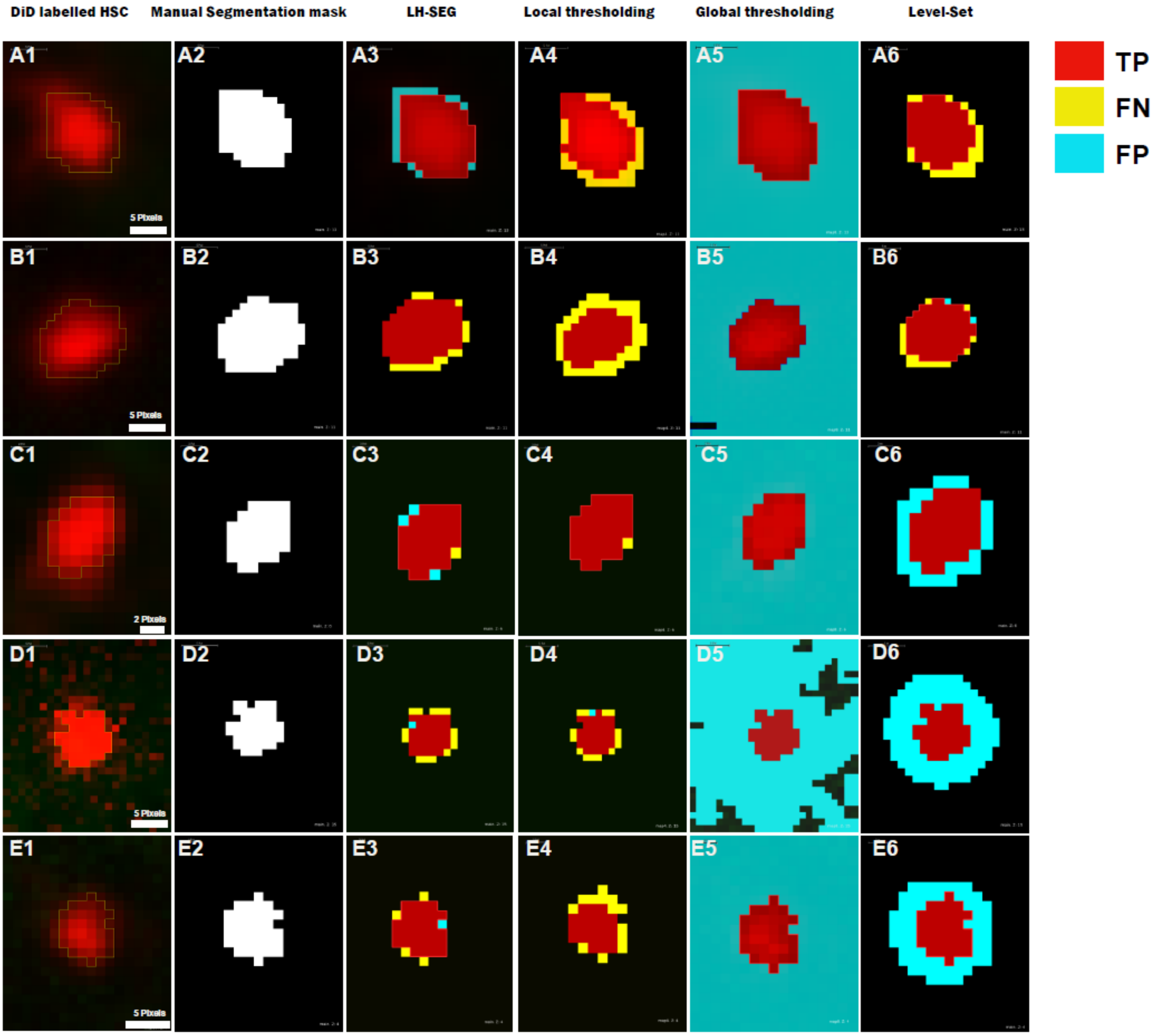
DiD labelled HSC
Manual Segmentation mask
LH-SEG
Local thresholding
Global thresholding
Level-Set
A1
A2
A3
A4
A5
A6
B1
B2
B3
B4
B5
B6
C1
C2
C3
C4
C5
C6
D1
D2
D3
D4
D5
D6
E1
E2
E3
E4
E5
E6
5 Pixels
5 Pixels
2 Pixels
5 Pixels
5 Pixels
TP
FN
FP

Figure S2.

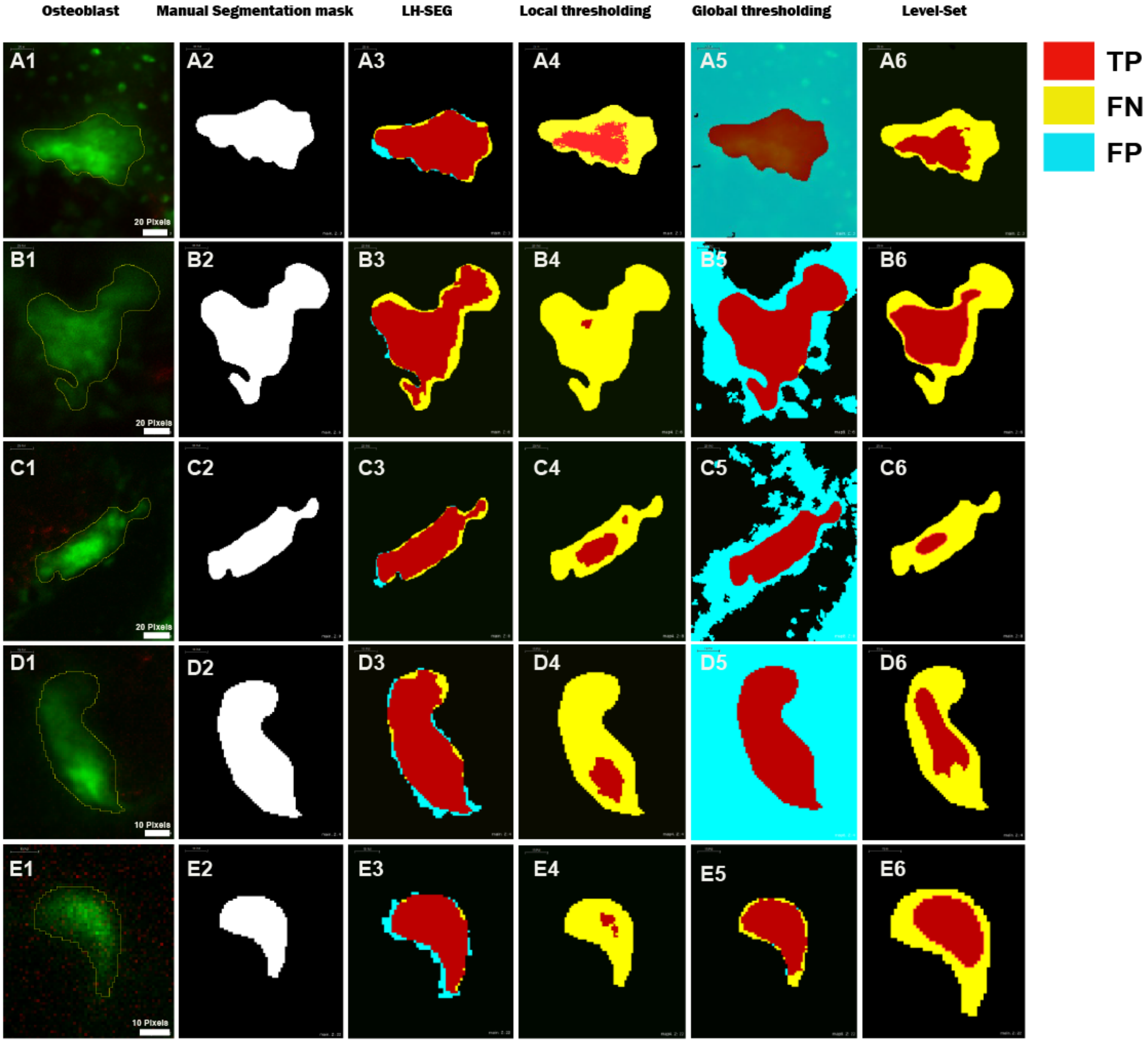

Osteoblast
Manual Segmentation mask
LH-SEG
Local thresholding
Global thresholding
Level-Set
TP
FN
FP
A1
A2
A3
A4
A5
A6
B1
B2
B3
B4
B5
B6
C1
C2
C3
C4
C5
C6
D1
D2
D3
D4
D5
D6
E1
E2
E3
E4
E5
E6
20 Pixels
20 Pixels
20 Pixels
10 Pixels
10 Pixels

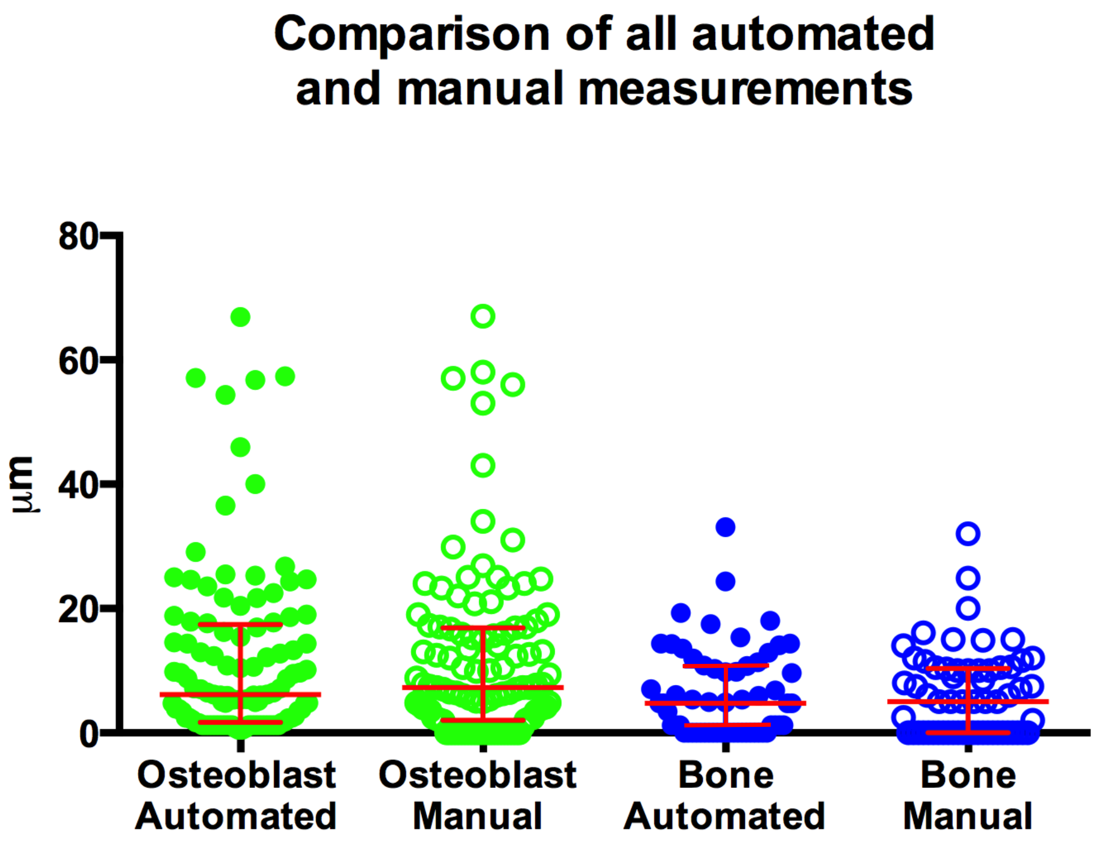
Comparison of all automated
and manual measurements
80
60
40
20
0
µm
Osteoblast
Automated
Osteoblast
Manual
Bone
Automated
Bone
Manual

Figure S1. Comparison of the manual benchmarking segmentation of DiD-labeled objects against the performance of our proposed Local Homogeneity segmentation method (LH-SEG), local adaptive thresholding, global thresholding and level set segmentation. (A1-E1) Examples of DiD-labeled objects exhibiting different intensity, edge morphology and neighborhood intensity characteristics. (A2-E2) Manual segmentation benchmarking masks of the DiD-labeled HSC. (A3 - E6) Segmentation masks obtained with the methods tested (ours (LH-SEG): A3-E3; local adaptive thresholding: A4-E4; global thresholding: A5-E5; level set segmentation: A6-E6). Red pixels are in both benchmark and tested mask (true positive TP); yellow pixels are in the benchmark but not in the test mask (false negative FN); blue pixels are in the test but not in the benchmark mask (false positive FP). The proportions of true positive, false positive and false negative pixels were used to evaluate the performance of each segmentation method. Pixel size is 0.8 µm for (A, B, D, E) and 1.2 µm for (C), Related to Performance evaluation; Segmentation accuracy section.

Figure S2. Comparison of the manual benchmarking segmentation of GFP$^+$ osteoblastic cells against the performance of our proposed Local Homogeneity segmentation method (LH-SEG), local adaptive thresholding, global thresholding and level set segmentation. (A1-E1) Examples of GFP$^+$ osteoblastic cells exhibiting different intensity, edges morphology and neighborhood intensity characteristics. (A2-E2) Manual segmentation benchmarking masks of the GFP$^+$ osteoblastic cells. (A3 - E6) Segmentation masks obtained with the methods tested (ours (LH-SEG): A3-E3; local adaptive thresholding: A4-E4; global thresholding: A5-E5; level set segmentation: A6-E6). Red pixels are in both benchmark and tested mask (true positive TP); yellow pixels are in the benchmark but not in the test mask (false negative FN); blue pixels are in the test but not in the benchmark mask (false positive FP). The proportions of true positive, false positive and false negative pixels were used to evaluate the performance of each segmentation method. Pixel size is 0.8 µm for (A, B, C, E) and 1.2 µm for (D), Related to Performance evaluation; Segmentation accuracy section.

Figure S3. Comparison of automated and manual (benchmark) 3D measurements. All identified HSCs across the 10 datasets were analyzed in 3D to identify the shorter distance between the cells and either osteoblasts (green) or bone (blue). In the graph, each circle represents a single analyzed cell. Automated measurements (full circles) and manual, benchmark measurements (empty circles) were very similar (see Figure 6 for error details) and provided a distribution patterns of ($p > 0.75$, Mann Whitney test; $n = 102$ measurements to

osteoblasts and 55 measurements to bone from 10 and 5 independent datasets, respectively). Related to Performance evaluation; 3D localization accuracy section.

Movie S1. Representative example of intravital microscopy data. A 3D stack containing DiD-labeled objects (red), GFP+ osteoblastic cells (green) and SHG signal of bone collagen fibers (blues) is shown as a z-series of 22 slices starting from top (bone) to bottom (deep bone marrow tissue). The z step size is 5 um, the field of view is 512 um2, Related to Results; Variability of Intravital Microscopy Datasets section.

Table S1. The 10 datasets used to develop and evaluate LH-SEG, HSC recognition and 3D measurements. Each dataset corresponds to a single mouse analysed by intravital microscopy and contains a variable number of 3D stacks (fields of view) acquired at varying magnification (213-620 in μm^2) and with step sizes of 1 or 5µm. All datasets included GFP and DiD signal an some included also second harmonic generation (SHG) signal from bone collagen. All datasets were from intravital microscopy of HSCs, however donor mice were either control (wilt type, WT) or infected (inf) animals, recipient mice were either wild type (WT) or col2.3Dkk1 (Dkk) transgenic, and the HSCs were FACS purified using either the LKS CD150 CD48 or LKS CD34 Flk2 combinations of markers, Related to Results; Variability of Intravital Microscopy Datasets section.

Table S2. Processing times and number of segments obtained for multi-resolution segmentation depending on the value of parameter α. Multi-resolution segmentation, the first step of LH-SEG, was run one the same 3D stack (dataset 10, field of view 4) using different values for parameter α for each channel (DiD signal, osteoblastic cells and bone collagen). Smaller α values led to larger numbers of segments and longer processing times, Related to Results; Local Heterogeneity-based Image Segmentation (LH-SEG) section.

Table S3. Summary of the optimized parameters used for LH-SEG. DiD, GFP osteoblast and SHG bone collagen signals required each their own set of optimized parameters, however the same parameters could be used for all the images throughout the datasets analysed. The first step of LH-SEG required optimization of parameter α, and the second step of LH-SEG required optimization of parameters T_{Δ_k} and d . The values selected for each parameter and

each channel/object are indicated, Related to Results; Local Heterogeneity-based Image Segmentation (LH-SEG) section.

Table S4. The intensity, morphological and textural features used to train the classifier. List of all features used to train the classifier, grouped by their type. The classifier itself selected the features highlighted in red as the discriminative ones to differentiate HSCs (in two classes) from non-specific DiD signal, Related to Results; Machine Learning Classification of HSCs section.

Segment-Classify-Measure-distance-3D.dcp is the source code file, ready to use in Definiens environment for quantifying HSCs, osteoblast and bone. For quantifying other bone marrow components follow the instructions provided in the supplemental data sections: Guidance on how to optimise LH-SEG parameters, Optimised LH-SEG parameter values for the experiments described and Table S3.

Table S1. The 10 datasets used to develop and evaluate LH-SEG, HSC recognition and 3D measurements.

Data type	Data set No.	Fields of view	Z (no. of Slices)	X,Y μm^2	Step size μm	Channels analysed	Type of experiment	HSCs Markers
3D Stack	1	12	37-88	213-396	1	DiD, GFP	WT into WT	LKS,CD150+, CD48-
3D Stack	2	9	62-92	248-378	1	DiD, GFP	WT into Dkk	LKS, CD150+, CD48-
3D Stack	3	10	45-94	268-405	1	DiD, GFP	WT into WT	LKS, CD150+, CD48-
3D Stack	4	8	57-102	308-450	1	DiD, GFP	WT into Dkk	LKS, CD150+, CD48-
3D Stack	5	10	14-29	620	5	DiD, GFP, SHG	WT into WT	LKS, CD34-, Flk2-
3D Stack	6	6	17-28	620	5	DiD, GFP, SHG	WT into WT	LKS, CCD34-, Flk2-
3D Stack	7	10	13-35	620	5	DiD, GFP	WT into WT	LKS, CD150+, CD48-
3D Stack	8	10	12-40	620	5	DiD, GFP,SHG	WT into WT	LKS, CD150+, CD48-
3D Stack	9	10	20-27	620	5	DiD, GFP.SHG	Inf into WT	LKS, CD34-, Flk2-
3D Stack	10	10	17-25	620	5	DiD, GFP,SHG	Inf into WT	LKS, CD34-, Flk2-

Table S2. Processing times and number of segments obtained for multi-resolution segmentation depending on the value of parameter α.

Cellular component	Scale parameter	Processing time	Number of segments
DiD labeled HSC	α = 3	01.08 mins	274.143
DiD labeled HSC	α = 8	00.37 mins	56,449
GFP+ osteoblastic cells	α = 3	8.47 mins	492 ,562
GFP+ osteoblastic cells	α = 10	1.06 mins	56,516
SHG bone collagen signal	α = 3	6.16.80 mins	501,339
SHG bone collagen signal	α = 8	01.09.30 mins	71,320

Table S3. Summary of the optimized parameters used for LH-SEG.

Object type	Multi-resolution optimized parameter	MDN optimized parameters	
HSCs	α =8	$T_{\bar{\Delta}_k} \geq 68$	$d = 30$
Osteoblast	α =10	$T_{\bar{\Delta}_k} \geq 8$	$d = 60$
Bone	α =8	$T_{\bar{\Delta}_k} \geq 4$	$d = 70$
Vasculature	α =8	$T_{\bar{\Delta}_k} \geq 4$	$d = 40$
Nestin	α =8	$T_{\bar{\Delta}_k} \geq 3$	$d = 60$
mTmG progenitors	α =8	$T_{\bar{\Delta}_k} \geq 10$	$d = 30$
Macrophages	α =8	$T_{\bar{\Delta}_k} \geq 68$	$d = 30$

Table S4. The intensity, morphological and textural features used to train the classifier. Features selected by the classifier as the most discriminative features are highlighted in red.

Feature	Feature type
Brightness	Intensity
Standard deviation	Intensity
Area	Morphological
Border length	Morphological
Length	Morphological
Length/width	Morphological
Number of pixels	Morphological
Rel. Border to image Border	Morphological
Thickness	Morphological
Volume	Morphological
Width	Morphological
Asymmetry	Morphological
Compactness	Morphological
Density	Morphological
Elliptic fit	Morphological
Radius of largest enclosed ellipse	Morphological
Radius of smallest enclosed ellipse	Morphological
Rectangular fit	Morphological
Roundness	Morphological
Shape index	Morphological
GLCM Homogeneity	Textural
GLCM Dissimilarity	Textural
GLCM Entropy	Textural
GLCM Ang. 2nd Moment	Textural
GLCM Mean	Textural
GLCM Std. Dev.	Textural
GLCM Correlation	Textural
GLDV Entropy	Textural
GLCM Contrast	Textural
GLCM StdDev	Textural

Guidance on how to optimise LH-SEG parameters:

In practice, users will have to select only three parameter for each object type (e.g. HSC, osteoblast, bone etc.) and each parameter would have to be optimized one time before starting the analysis, once optimized it should work for different image datasets given that the object types are still the same.

For each parameter users only need to provide a rough estimate of the average parameter value. The parameters are then integrated into the algorithm automatically.

Parameter 1: Multi-resolution parameter α

This parameter is mainly responsible for the selection of the segments size resulting from applying the Multi-resolution segmentation. Higher values for the scale parameter will result in larger image segments, while smaller values will result in smaller image segments. The default setting for this parameter is 8 and users are advised to use it. Smaller values will result in increased running time. A good indication to confirm the correct parameter selection will be edge detection of the regions of interests.

Parameter 2: Mean intensity difference to neighbour threshold $T_{\bar{\Delta}_k}$

The value of the threshold along with the neighbourhood size (Parameter 2) will determine which segments (resulted from the multi-resolution) are merged together to form the real edges of the object of interest. To select the parameter $T_{\bar{\Delta}_k}$ value, the users need to provide a rough estimate of the mean intensity difference to neighbour threshold. To do this the user can activate the mean intensity difference to neighbour feature available in the feature window of Definiens, select segments from the background that are close to the edges of the object of interest then select the highest value found and set it as a threshold.

Parameter 3: neighbourhood size d

The selection of the parameter d determines the size of the neighbourhood in which a particular object covers. To select a neighbourhood size, the users need to provide a rough estimate of the average object diameter size in pixels.

Optimised LH-SEG parameter values for the experiments described

- Parameter optimization for the multi-resolution segmentation parameter α for each image object category: To detect small objects such as HCSs, bone marrow micro-cavities, myeloid progenitors, nestin GFP cells and microvessels we selected a slightly smaller scale parameter $\alpha = 8$ compared to that used for larger objects such as osteoblasts $\alpha = 10$. Importantly, selecting smaller scale parameters did not result in significant edge detection improvement, but did increase computational time thus providing support for the values chosen above.

- Optimization of parameter $T_{\bar{\Delta}_k}$ to correctly segment each image object category MDN: a high threshold of $T_{\bar{\Delta}_k} \geq 68$ for HSCs and macrophages was selected to restrict the selection of segments to those with higher intensities, in contrast we select smaller threshold for tomato expressing progenitors $T_{\bar{\Delta}_k} \geq 10$ osteoblast $T_{\bar{\Delta}_k} \geq 8$, bone and vasculature $T_{\bar{\Delta}_k} \geq 4$ and nestin $T_{\bar{\Delta}_k} \geq 3$ to allow the selection of segment with more heterogonous intensities.

- Optimization of parameter d to correctly segment each image object category: for the HCSs d was based on our observation that a single HSC will most likely cover a neighborhood of 10-30 pixels, depending on the HSC size (8-12µm in diameter) (Lo Celso et al., 2009; Lo Celso et al., 2011) and the magnification settings used, therefore we set $d = 30$. The same was for progenitors and macrophages. In contrast, given that osteoblasts and bone occupy relatively larger neighborhoods, we set $d = 60$ for the osteoblast and nestin cells $d = 70$ for bone collagen and $d = 40$ for vessels.

Definition of each selected feature by the decision tree classifier

1) Roundness

The Roundness feature describes how much the shape of an image object is similar to an ellipsoid. The more the shape of an image object is similar to an ellipsoid, the lower its roundness. It is calculated by the difference of the enclosing ellipsoid and the enclosed ellipsoid. The radius of the largest enclosed ellipsoid is subtracted from the radius of the smallest enclosing ellipsoid.

2) Length/width

The length-to-width ratio of an image object in 3D

3) Grey level co-occurrence matrix (GLCM) contrast

Contrast is the opposite of homogeneity. It is a measure of the amount of local variation in the image. It increases exponentially as i, j increase.

$$\sum_{i,j=0}^{N-1} P_{i,j}(i-j)^2$$

Where i is the row number, j is the column number, $P_{i,j}$ is the normalized value in the cell and N is the number of rows or columns.

As defined in the Defineins reference book. For more information on the parameters used to calculate the features refer to the Defineins reference book.
